# A Class of Robust Estimators for Moment Condition Models

**DOI:** 10.3390/e28070828

**Published:** 2026-07-21

**Authors:** Amor Keziou, Aida Toma

**Affiliations:** 1 Laboratoire de Mathématiques de Reims (LMR, UMR CNRS 9008), Université de Reims Champagne-Ardenne, UFR SEN, Moulin de la Housse, B.P. 1039, 51687 Reims, France; amor.keziou@univ-reims.fr; 2 Department of Applied Mathematics, Bucharest University of Economic Studies, Piaţa Romană no. 6, 010374 Bucharest, Romania; 3 “Gheorghe Mihoc-Caius Iacob” Institute of Mathematical Statistics and Applied Mathematics of the Romanian Academy, Calea 13 Septembrie no. 13, 050711 Bucharest, Romania

**Keywords:** moment condition models, estimation, robustness, divergence measures

## Abstract

Moment condition models are popular in statistics and econometrics, as they provide a powerful and flexible framework for estimation. However, estimation procedures based on these models can be sensitive to misspecification or the presence of outliers in the data. In the present paper, we introduce a class of robust estimators for moment condition models, representing robust alternatives to minimum empirical divergence estimators. The estimators are constructed by using truncated orthogonality functions and minimizing divergences in dual form, allowing to limit the impact of outliers or model deviations. We give the expressions of the influence functions of the estimators and prove their robustness. We also prove that the estimators are consistent. These theoretical results together with numerical examples, based on Monte Carlo simulations, show that extreme observations do not disproportionately affect the final estimates.

## 1. Introduction

In many statistical applications, models are not fully specified through a parametric distribution, but instead they are defined through a set of moment conditions. A moment condition model is a family $M^{\left(1\right)}$ of probability measures (p.m.), all defined on the same measurable space $\left(R^{m},B\left(R^{m}\right)\right)$, such that (s.t.)

(1) ∫g(x,θ)dQ(x)=0,

for all $Q\in M^{\left(1\right)}$. The parameter of interest θ belongs to some subset $\Theta \subset R^{d}$. The function $g:={\left(g_{1},\ldots ,g_{\ell }\right)}^{T}$, with ℓ≥d, is defined on $R^{m}\times \Theta$, with each of the $g_{i}$’s being real valued. Denote by $M^{\left(1\right)}$ the set of all p.m. on $\left(R^{m},B\left(R^{m}\right)\right)$, and for each θ∈Θ, define

$$M_{\theta }^{\left(1\right)}:=\left\{Q\in M^{\left(1\right)}\;s.t.\int g\left(x,\theta \right)\;dQ\left(x\right)=0\right\},$$

so that

$$M^{\left(1\right)}=\underset{\theta \in \Theta }{⋃}M_{\theta }^{\left(1\right)}.$$

Moment condition models are popular in statistics and econometrics, as they provide a powerful and flexible framework for estimation (see [1,2]).

Consider the problem of estimating the unknown true value of the parameter $\theta_{0}$ for which

$$\int g\left(x,\theta_{0}\right)\;dP_{0}\left(x\right)=0,$$

$\theta_{0}$ being the unique solution of the equation. The functions $g_{1}\left(\cdot ,\theta \right),\ldots ,g_{\ell }\left(\cdot ,\theta \right),1_{R^{m}}$ are supposed to be linearly independent, in the sense that $P_{0}\left(x\in R^{m}/t_{0}+\sum_{j=1}^{l}t_{j}g_{j}\left(x,\theta \right)\neq 0\right)>0$, for all $t\in R^{l+1}$, t≠0.

Among the classical approaches, well known in the literature, we recall the Generalized Method of Moments (GMM) proposed in [3], the empirical likelihood (EL) estimator (see [2]), the continuous updated (CU) estimator [4], the exponential tilting (ET) estimator [5], and the generalized empirical likelihood (GEL) estimators [6]. Under standard regularity conditions, GMM estimators are consistent, asymptotically normal, and efficient. The EL estimator is superior to the previous ones in terms of higher-order asymptotic efficiency, but this property holds only under correct specification of the moment conditions. The GEL class of estimators extends EL and CU by using alternative divergence measures. They often provide better finite-sample performance than GMM, producing more accurate estimates. Unlike EL, the GEL estimators are more flexible, allowing alternative divergence functions that improve numerical stability.

Despite their appealing asymptotic properties, all the estimators mentioned above face several challenges in practice. If the moment conditions are misspecified or the data set contains outliers, then these estimators may lead to erroneous results. This drawback naturally led to the development of robust estimation methods, which modify moment conditions or objective functions to achieve greater stability in empirical applications. Among the robust alternatives known in the literature, we recall the robust version of GMM estimators [7], the robust empirical tilted estimator [8], as well as a robust versions of EL proposed in [9].

A general methodology for estimation and testing in moment condition models has been proposed in [10]. This approach is based on minimizing divergences in their dual form and allows the asymptotic study of the estimators, called minimum empirical divergence estimators, and of the associated test statistics, both under the model and under misspecification of the model. The broad class of minimum empirical divergence estimators provides a general framework for estimation in moment condition models, encompassing the EL estimator, the ET estimator, and the CU estimator as special cases. These estimators are well known to achieve first-order efficiency under correct specification of the model and to enjoy a unified divergence-based formulation. Despite these appealing efficiency properties, their robustness characteristics remain limited, since they inherit the full sensitivity of the original moment conditions. Their influence functions are unbounded, and consequently, even a small amount of contamination can have an arbitrarily large effect on these estimators.

In this paper, we propose a robust extension of the minimum empirical divergence framework. The construction is based on truncating the orthogonality conditions via a Huber-type transformation and performing minimization of empirical divergences in their dual representation. This modification yields estimations with controlled sensitivity to extreme observations while preserving the structure of the original divergence framework. A key result of the paper is that, in contrast to the original minimum empirical divergence estimators, the proposed estimators possess bounded influence functions. This provides a formal robustness guarantee and establishes resistance to infinitesimal contamination. The estimators are also consistent. These theoretical results, together with numerical examples, based on Monte Carlo simulations, show that extreme observations do not disproportionately affect the final estimates.

The proposed framework generates a broad class of robust estimators indexed by the choice of the divergence. This framework bridges the gap between efficiency-driven empirical divergence methods and robustness considerations, providing a general class of estimators with bounded influence. We formally show that, for particular divergences, the proposed methodology recovers as special cases a robust version of the empirical likelihood estimator and the robust exponential tilting estimator previously introduced in the literature. The proposed framework provides a unified theoretical formulation for these robust estimators. Such a formulation makes it possible to study their properties within a common mathematical setting, to derive new robust estimators through alternative divergence choices, and to investigate structural properties, such as equivariance, at the level of the entire class. In particular, since minimum empirical divergence estimators possess well-established equivariance properties, the present framework opens the possibility of extending these results to the robust estimators introduced here and of investigating minimum-risk equivariant robust estimation within this class. These questions constitute a natural direction for our future research. All these aspects motivated our studies in the present paper.

## 2. Robust Minimum Empirical Divergence Estimators

Divergences between probability measures are widely used in statistics and data science in order to perform inference in models of various kinds, parametric or semiparametric. Statistical methods based on divergence minimization extend the likelihood paradigm and often have the advantage to provide a trade-off between efficiency and robustness. The approach based on minimizing dual forms of divergences was initially used in the case of parametric models, with the results being published in a series of articles [11,12]. In the framework of moment condition models, this approach was considered to define minimum empirical divergence estimators in [10]. In this section, we define a class of estimators for moment condition models, representing robust alternatives to minimum empirical divergence estimators. We prove robustness properties by the means of the influence function approach, as well the consistency of these estimators. All the proofs of the theoretical results contained in the paper are deferred in Appendix A.

An intuitive way to view the procedure is as a two-stage mechanism: truncation of the orthogonality conditions and estimation based on divergence minimization. The starting point is the observation that the lack of robustness of classical minimum empirical divergence estimators originates from the moment conditions themselves. In the standard framework, each observation contributes to the estimation through the original orthogonality functions. When an observation is extreme, its contribution can become disproportionately large, allowing a small number of atypical observations to dominate the estimation procedure. To prevent this effect, we replace the original orthogonality functions by truncated versions constructed using the multivariate Huber function. Observations that are consistent with the model are essentially left unchanged, whereas observations that generate excessively large discrepancies are prevented from exerting unlimited influence. Once the orthogonality conditions have been robustified, estimation proceeds through the minimization of an empirical divergence in its dual form. Whereas truncation controls sensitivity to extreme observations, divergence minimization determines how the information contained in the sample is aggregated to estimate the parameter of interest.

### 2.1. Statistical Divergences

Let φ be a convex function defined on R and $\left[0,\infty \right]$ valued, with φ(1)=0, such that its domain

domφ:={x∈Rsuchthatφ(x)<∞}:=(a,b)

is an interval with endpoints a<1<b. We assume that φ is closed in the sense that if *a* or *b* is finite then φ(x)→φ(a) when x↓a, and φ(x)→φ(b) when x↑b. Let $P\in M^{\left(1\right)}$ be some probability measure. For any signed finite measure *Q* defined on the same measurable space $\left(R^{m},B\left(R^{m}\right)\right)$, absolutely continuous (a.c.) with respect to (w.r.t.) *P*, the φ-divergence between *Q* and *P* is defined by

(2) $$D_{\varphi }\left(Q,P\right):=\int \varphi \left(\frac{dQ}{dP}\left(x\right)\right)dP\left(x\right).\;$$

When *Q* is not a.c. with respect to P, we set $D_{\varphi }\left(Q,P\right)=\infty$. This definition extends the one of divergences between probability measures as given in [13].

The φ-divergence between some set Ω of signed finite measures and a probability measure *P* is defined by

(3) $$D_{\varphi }\left(\Omega ,P\right)=\underset{Q\in \Omega }{\inf}D_{\varphi }\left(Q,P\right).$$

For example, the class of power divergences, introduced in [14], is defined by the convex functions

(4) $$x\in R_{+}^{*}\mapsto \varphi_{\gamma }\left(x\right):=\frac{x^{\gamma }-\gamma x+\gamma -1}{\gamma (\gamma -1)},$$

for γ∈R∖{0,1} and $x\in R_{+}^{*}\mapsto \varphi_{0}\left(x\right):=\lim_{\gamma \to 0}\varphi_{\gamma }\left(x\right)=-\log x+x-1$, $x\in R_{+}^{*}\mapsto \varphi_{1}\left(x\right):=\lim_{\gamma \to 1}\varphi_{\gamma }\left(x\right)=x\log x-x+1$. Some well-known divergences belong to this class. The Kullback–Leibler (KL) divergence is associated with $\varphi_{1}$, the modified Kullback–Leibler (K$L_{m}$) with $\varphi_{0}$, the $\chi^{2}$ divergence with $\varphi_{2}$, the modified $\chi^{2}$ divergence with $\varphi_{-1}$ and the Hellinger distance with $\varphi_{1/2}$. When $\varphi_{\gamma }$ is not defined on (−∞,0) or when $\varphi_{\gamma }$ is not convex, the definition of the corresponding power divergence function $Q\in M^{\left(1\right)}\mapsto D_{\varphi_{\gamma }}\left(Q,P\right)$ can be extended on the whole set of signed finite measures by taking the following extension of $\varphi_{\gamma }$

$$\varphi_{\gamma }:\;x\in R\mapsto \varphi_{\gamma }\left(x\right)1_{\left[0,\infty \right)}\left(x\right)+\left(+\infty \right)1_{\left(-\infty ,0\right)}\left(x\right).\;$$

Throughout the paper, we assume that φ is strictly convex on its domain (a,b) and twice continuously differentiable on the interior of the domain. Then $\varphi^{\prime }\left(1\right)=0$ and $\varphi^{\prime }$ is strictly increasing, so it is injective and consequently invertible on its image. The functions $\varphi_{\gamma }$, corresponding to the power divergences, satisfy the above conditions. We will make use of the convex conjugate ψ of the function φ defined by

(5) $$\psi \left(u\right)=\underset{x\in R}{\sup}\;\left\{ux-\varphi (x)\right\},\forall u\in R.\;$$

Under the above conditions on the function φ, see e.g., ref. [15], it holds that the function ψ is strictly convex (on its domain) and closed; its domain is an interval $\left(a^{*},b^{*}\right)$, with endpoints satisfying $a^{*}<0<b^{*}$, with $a^{*}=\lim_{x\to -\infty }\frac{\varphi (x)}{x}=\lim_{x↓a}\frac{\varphi (x)}{x}=\lim_{x↓a}\varphi^{\prime }\left(x\right)$ and $b^{*}=\lim_{x\to \infty }\frac{\varphi (x)}{x}=\lim_{x↑b}\frac{\varphi (x)}{x}=\lim_{x↑b}\varphi^{\prime }\left(x\right).$ Hence, we have

(6) $$\psi \left(u\right)=\left\{\begin{array}{cc} u{\varphi^{\prime }}^{-1}\left(u\right)-\varphi \left({\varphi^{\prime }}^{-1}\left(u\right)\right), & \forall u\in ]a^{*},b^{*}[;\\ \lim_{u↓a^{*}}u{\varphi^{\prime }}^{-1}\left(u\right)-\varphi \left({\varphi^{\prime }}^{-1}\left(u\right)\right), & \mathrm{if}\;u=a^{*};\\ \lim_{u↑b^{*}}u{\varphi^{\prime }}^{-1}\left(u\right)-\varphi \left({\varphi^{\prime }}^{-1}\left(u\right)\right), & \mathrm{if}\;u=b^{*};\\ \infty ,\;\;\forall u\notin [a^{*},b^{*}]. \end{array}\right.\;$$

### 2.2. Definition of the Estimators

We consider a reference model $\left\{P_{\theta };\theta \in \Theta \right\}$, such that, for each θ∈Θ, the probability measure $P_{\theta }$ belongs to $M_{\theta }$; hence, $\int g\left(x,\theta \right)\;dP_{\theta }\left(x\right)=0$ and θ is the unique solution of the equation. $\left\{P_{\theta };\;\theta \in \Theta \right\}$ is not necessarily a parametric model; the index θ of $P_{\theta }$ indicates only that $P_{\theta }\in M_{\theta }$. The p.m. $P_{0}$, here denoted $P_{\theta_{0}}$, corresponding to the unknown parameter $\theta_{0}$ to be estimated, belongs to this reference model. This reference model will be associated with the truncated orthogonality function used to define new robust estimators of $\theta_{0}$.

We consider the function $g^{c}:R^{m}\times \Theta \to R^{\ell }$,

(7) $$g^{c}\left(x,\theta \right):=H_{c}\left(A\left(\theta ,P_{\theta }\right)\;\left[g\left(x,\theta \right)-\tau \left(\theta ,P_{\theta }\right)\right]\right),\;$$

where $H_{c}:R^{\ell }\to R^{\ell }$ is Huber’s function

(8) $$H_{c}\left(y\right):=\left\{\begin{array}{c} y\cdot \min\left(1,\frac{c}{∥y∥}\right)\;\mathrm{if}\;y\neq 0,\\ 0\;\mathrm{if}\;y=0,\; \end{array}\right.$$

matrix $A(\theta ,P_{\theta })$ and vector $\tau (\theta ,P_{\theta })$ are defined by the solutions of the system

(9) $$\left\{\begin{array}{c} \int g^{c}\left(x,\theta \right)\;dP_{\theta }\left(x\right)=0,\\ \int g^{c}\left(x,\theta \right)\;g^{c}{\left(x,\theta \right)}^{⊤}\;dP_{\theta }\left(x\right)=I_{\ell }, \end{array}\right.\;$$

where $I_{\ell }$ is the ℓ×ℓ identity matrix and *c* is a given positive constant. We also use the auxiliary function $h^{c}\left(x,\theta ,A,\tau \right):=H_{c}\left(A\;\left[g\left(x,\theta \right)-\tau \right]\right)$ when we need to work with the dependence on matrix *A* and on vector τ. Then

(10) $$h^{c}\left(x,\theta ,A\left(\theta ,P_{\theta }\right),\tau \left(\theta ,P_{\theta }\right)\right)=g^{c}\left(x,\theta \right).\;$$

For given $P_{\theta }\in M_{\theta }$, the triplet $\left(\theta ,A\left(\theta ,P_{\theta }\right),\tau \left(\theta ,P_{\theta }\right)\right)$ is the unique solution of the system

(11) $$\left\{\begin{array}{c} \int g\left(x,\theta \right)\;dP_{\theta }\left(x\right)=0,\\ \int g^{c}\left(x,\theta \right)\;dP_{\theta }\left(x\right)=0,\\ \int g^{c}\left(x,\theta \right)\;g^{c}{\left(x,\theta \right)}^{⊤}\;dP_{\theta }\left(x\right)=I_{\ell }. \end{array}\right.\;$$

The construction of the function $g^{c}\left(x,\theta \right)$, as well as the uniqueness of the solution $\left(\theta ,A\left(\theta ,P_{\theta }\right),\tau \left(\theta ,P_{\theta }\right)\right)$ for the system (11), are presented and justified in [7], (p. 48). According to those results, the uniqueness of the solution is implied by the implicit function theorem and the Fréchet differentiability of the equation system defining $\left(\theta ,A\left(\theta ,P_{\theta }\right),\tau \left(\theta ,P_{\theta }\right)\right)$ in a neighborhood of $P_{\theta }$, which itself is implied by the boundedness of the function $g^{c}$.

Consider the problem of estimating the triplet $\left(\theta_{0},A\left(\theta_{0},P_{\theta_{0}}\right),\tau \left(\theta_{0},P_{\theta_{0}}\right)\right)$ on the basis of an i.i.d. sample $X_{1},\ldots ,X_{n}∼P_{\theta_{0}}$, $P_{\theta_{0}}\in M_{\theta_{0}}$. For each θ∈Θ, using the p.m. $P_{\theta }$ from the reference model, we define $A_{n}\left(\theta \right)$ and $\tau_{n}\left(\theta \right)$ solutions of

(12) $$\left\{\begin{array}{c} \int h^{c}\left(x,\theta ,A_{n}\left(\theta \right),\tau_{n}\left(\theta \right)\right)\;dP_{\theta }\left(x\right)=0,\\ \int h^{c}\left(x,\theta ,A_{n}\left(\theta \right),\tau_{n}\left(\theta \right)\right)h^{c}{\left(x,\theta ,A_{n}\left(\theta \right),\tau_{n}\left(\theta \right)\right)}^{⊤}\;dP_{n}\left(x\right)=I_{\ell }, \end{array}\right.\;$$

$P_{n}\left(\cdot \right):=\frac{1}{n}\sum_{i=1}^{n}\delta_{X_{i}}\left(\cdot \right)$ is the empirical measure associated with the sample, with $\delta_{x}\left(\cdot \right)$ being the Dirac measure at point *x*, for any *x*. Then we define

(13) $$g_{n}^{c}\left(x,\theta \right):=h^{c}\left(x,\theta ,A_{n}\left(\theta \right),\tau_{n}\left(\theta \right)\right)=H_{c}\left(A_{n}\left(\theta \right)\;\left[g\left(x,\theta \right)-\tau_{n}\left(\theta \right)\right]\right).\;$$

Consider the moment condition model associated with the function $g_{n}^{c}\left(x,\theta \right)$, namely,

(14) $$M^{c,n}:=\underset{\theta \in \Theta }{⋃}M_{\theta }^{c,n},\;$$

where

(15) $$M_{\theta }^{c,n}:=\left\{Q\in M\;s.t.\;\int g_{n}^{c}\left(x,\theta \right)\;dQ\left(x\right)=0\right\},\;\forall \theta \in \Theta$$

and *M* is the set of all signed finite measures defined on $\left(R^{m},B\left(R^{m}\right)\right)$. For a given φ-divergence, a given θ∈Θ and a given p.m. *P*, define the set

$$\Lambda_{\theta }^{c,n}\left(P\right):=\left\{t:={\left(t_{0},t_{1},\ldots ,t_{\ell }\right)}^{⊤}\in R^{1+\ell }\;s.t.\;\int \left|\psi \left(t^{⊤}\;{\bar{g}}_{n}^{c}\left(x,\theta \right)\right)\right|\;dP\left(x\right)<\infty \right\},\;$$

where ψ(·) is the convex conjugate of φ(·), given by (6), and ${\bar{g}}_{n}^{c}:={\left(1_{R^{m}},{g_{n}^{c}}^{⊤}\right)}^{⊤}$. Denote $\Lambda_{\theta }^{c,n}:=\Lambda_{\theta }^{c,n}\left(P_{\theta_{0}}\right)$ and $\Lambda_{\theta }^{c,n,n}:=\Lambda_{\theta }^{c,n}\left(P_{n}\right).$ Since $g_{n}^{c}\left(x,\theta \right)$ is bounded with respect to *x*, if we assume that $D_{\varphi }\left(M_{\theta }^{c,n},P_{\theta_{0}}\right)$ is finite, then on the basis of Corollary 1.2 from [16], the following dual representation holds

(16) $$D_{\varphi }\left(M_{\theta }^{c,n},P_{\theta_{0}}\right)=\underset{t\in \Lambda_{\theta }^{c,n}}{\sup}\int m_{n}^{c}\left(x,\theta ,t\right)\;dP_{\theta_{0}}\left(x\right),\;$$

where $m_{n}^{c}\left(x,\theta ,t\right):=t_{0}-\psi \left(t^{⊤}{\bar{g}}_{n}^{c}\left(x,\theta \right)\right)$. Supremum in (16) is achieved in $t_{\theta }^{c,n}:=t_{\theta }^{c,n}\left(P_{\theta_{0}}\right)$, defined by

(17) $$t_{\theta }^{c,n}:=\underset{t\in \Lambda_{\theta }^{c,n}}{\arg\sup}\;\int m_{n}^{c}\left(x,\theta ,t\right)\;dP_{\theta_{0}}\left(x\right).$$

According to Proposition 4.2 from [10], for each θ∈Θ, the condition

(18) $$P_{\theta_{0}}\left(\{x\in R^{m}\;s.t.\;t^{⊤}\;{\bar{g}}_{n}^{c}\left(x,\theta \right)\neq 0\}\right)>0,\;\;\mathrm{for}\;\mathrm{all}\;t\in R^{1+\ell }\setminus \left\{0\right\},$$

ensures that $t_{\theta }^{c,n}$ defined as a solution of the optimization problem (17) is unique. Note that the linear independence of the functions $1_{R^{m}},g_{n,1}^{c}\left(\cdot ,\theta \right),\ldots ,g_{n,\ell }^{c}\left(\cdot ,\theta \right)$ implies condition (18), whenever $P_{\theta_{0}}$ is not degenerate. A natural estimator of $t_{\theta }^{c,n}$ is defined by

(19) $${\hat{t}}_{\theta }^{c}:=\underset{t\in \Lambda_{\theta }^{c,n,n}}{\arg\sup}\;\int m_{n}^{c}\left(x,\theta ,t\right)\;dP_{n}\left(x\right).\;$$

Then, a plug-in estimator of $D_{\varphi }\left(M_{\theta }^{c,n},P_{\theta_{0}}\right)$ is

(20) $${\hat{D}}_{\varphi }\left(M_{\theta }^{c,n},P_{\theta_{0}}\right):=\underset{t\in \Lambda_{\theta }^{c,n,n}}{\sup}\int m_{n}^{c}\left(x,\theta ,t\right)\;dP_{n}\left(x\right).\;$$

Finally, an estimator of the parameter $\theta_{0}$ is defined by

(21) $${\hat{\theta }}_{\varphi }^{c}:=\underset{\theta \in \Theta }{\arg\inf}\underset{t\in \Lambda_{\theta }^{c,n,n}}{\sup}\;\int m_{n}^{c}\left(x,\theta ,t\right)\;dP_{n}\left(x\right).\;$$

The proposed framework provides a unified formulation for constructing robust estimators in moment condition models based on truncated orthogonality functions and dual forms of divergences. The new class of estimators encompasses several existing robust estimators as particular cases. From the general estimation procedure, these estimators are obtained through appropriate choices of the divergence. In Remark 1, the robust empirical tilting estimator from [8] and a robust version of the EL estimator are shown to arise as special cases when the KL divergence and the $KL_{m}$ divergence, respectively, are used in the proposed procedure.

**Remark** **1. ** *(a) For the particular choice of KL divergence, the above estimator (21) can be written as*

(22) $${\hat{\theta }}_{\varphi_{1}}^{c}:=\underset{\theta \in \Theta }{\arg\inf}\underset{t\in \Lambda_{\theta }^{c,n,n}}{\sup}\;\left\{t_{0}-\int \exp\left(t^{⊤}{\bar{g}}_{n}^{c}\left(x,\theta \right)\right)-1\;dP_{n}\left(x\right)\right\},\;$$

*which coincides with the robust empirical tilting estimator from [8].*

*(b) Choosing the $KL_{m}$ divergence in the above expressions, we obtain*

(23) $${\hat{\theta }}_{\varphi_{0}}^{c}:=\underset{\theta \in \Theta }{\arg\inf}\underset{t\in \Lambda_{\theta }^{c,n,n}}{\sup}\;\left\{\int \bar{\log}\left(1-t^{⊤}g_{n}^{c}\left(x,\theta \right)\right)\;dP_{n}\left(x\right)\right\},\;$$

*where, here,*

$$\Lambda_{\theta }^{c,n,n}=\left\{t\in R^{\ell }\;s.t.\;\int \left|\bar{\log}\left(1-t^{⊤}g_{n}^{c}\left(x,\theta \right)\right)\right|\;dP_{n}\left(x\right)<\infty \right\},$$

*and $\bar{\log}\left(\cdot \right)$ is the extended logarithm function, defined by*

$$\bar{\log}\left(x\right)=\left\{\begin{array}{ccc} \log(x) & if & x>0;\\ -\infty & if & x\leq 0. \end{array}\right.\;$$

*The estimator (23) can be seen as a robust version of the EL one. This is because the classical EL estimator can be written as (see, e.g., ref. [1])*

(24) $$\hat{\theta }=\underset{\theta \in \Theta }{\arg\inf}\;\underset{\left(t_{1},\ldots ,t_{\ell }\right)\in R^{\ell }}{\sup}\;\left\{\frac{1}{n}\sum_{i=1}^{n}\bar{\log}\left(1-t^{⊤}g\left(x,\theta \right)\right)\right\},\;$$

*while the estimator ${\hat{\theta }}_{\varphi_{0}}^{c}$, having a similar expression to that of $\hat{\theta }$ in which g is replaced with the bounded function $g_{n}^{c}$, is robust, according to the results obtained in Section 2.3.*

*(c) Choosing the $\chi^{2}$-divergence, we obtain the following robust version of the empirical $\chi^{2}$-divergence estimator*

(25) $${\hat{\theta }}_{\varphi_{2}}^{c}:=\underset{\theta \in \Theta }{\arg\inf}\underset{t\in \Lambda_{\theta }^{c,n,n}}{\sup}\;\left\{t_{0}-\int \frac{1}{2}{\left(t^{⊤}{\bar{g}}_{n}^{c}\left(x,\theta \right)\right)}^{2}+t^{⊤}{\bar{g}}_{n}^{c}\left(x,\theta \right)\;dP_{n}\left(x\right)\right\}.\;$$

*Notice that, unlike the case of the above estimators, the supremum in (25) can be explicitly computed; in fact, it is reached in*

(26) $${\hat{t}}_{\theta }^{c}={\left[\int {\bar{g}}_{n}^{c}\left(x,\theta \right)\;{\bar{g}}_{n}^{c}{\left(x,\theta \right)}^{⊤}\;dP_{n}\left(x\right)\right]}^{-1}\;\left[\begin{array}{c} 0\\ \int g_{n}^{c}\left(x,\theta \right)\;dP_{n}\left(x\right) \end{array}\right].$$

### 2.3. Robustness Properties

We present robustness properties of the estimators ${\hat{t}}_{\theta }^{c}$ and ${\hat{\theta }}_{\varphi }^{c}$, defined respectively by (19) and (21), using the influence function approach. We recall that the influence function of the statistical functional *T* corresponding to an estimator ${\hat{\theta }}_{n}=T\left(P_{n}\right)$ measures the standardized effect of an infinitesimal contamination in a point on the asymptotic value of an estimator and is defined by

$$\mathrm{IF}\left(x;T,P_{0}\right):={\left.\frac{\partial }{\partial \varepsilon }T\left({\tilde{P}}_{\varepsilon x}\right)\right|}_{\varepsilon =0},\;$$

where ${\tilde{P}}_{\varepsilon x}=\left(1-\varepsilon \right)\;P_{0}+\varepsilon \;\delta_{x}$, ε∈]0,1[, with $\delta_{x}$ being the Dirac measure with all mass at *x*. Whenever the influence function is bounded with respect to *x*, the corresponding estimator is robust (see, e.g., ref. [17]).

An unbounded influence function implies that the effect of contamination is not controlled, such that the impact of a single observation can be arbitrarily large. In the framework of our procedure, truncation acts as an automatic weighting mechanism. Regular observations contribute fully to estimation, whereas extreme observations receive a limited influence. From the perspective of Hampel’s infinitesimal robustness theory, bounded influence functions guarantee that local departures from the assumed model cannot produce arbitrarily large perturbations of the estimator. Consequently, the estimators with bounded influence function are less sensitive to contamination, data errors, and model deviations. From a practical standpoint, this leads to more stable estimation and inference.

For given p.m. *P*, the statistical functional corresponding to the estimator ${\hat{t}}_{\theta }^{c}$ is defined by

(27) $$t_{\theta }^{c}\left(P\right):=\underset{t\in \Lambda_{\theta }^{c}\left(P\right)}{\arg\sup}\;\int m^{c}\left(x,\theta ,t,P\right)\;dP\left(x\right),\;$$

where

(28) $$\Lambda_{\theta }^{c}\left(P\right):=\left\{t\in R^{1+\ell }\;s.t.\;\int \left|\psi \left(t^{⊤}\;{\bar{g}}^{c}\left(x,\theta ,P\right)\right)\right|\;dP\left(x\right)<\infty \right\},$$

${\bar{g}}^{c}:={\left(1_{R^{m}},{g^{c}}^{⊤}\right)}^{⊤}$ and $g^{c}\left(x,\theta ,P\right):=h^{c}\left(x,\theta ,A\left(\theta ,P\right),\tau \left(\theta ,P\right)\right)$, with A(θ,P) and τ(θ,P) solutions of the system

(29) $$\left\{\begin{array}{c} \int h^{c}\left(x,\theta ,A\left(\theta ,P\right),\tau \left(\theta ,P\right)\right)\;dP_{\theta }\left(x\right)=0\\ \int h^{c}\left(x,\theta ,A\left(\theta ,P\right),\tau \left(\theta ,P\right)\right)\;h^{c}{\left(x,\theta ,A\left(\theta ,P\right),\tau \left(\theta ,P\right)\right)}^{⊤}\;dP\left(x\right)=I_{\ell } \end{array}\right.\;$$

and

(30) $$m^{c}\left(x,\theta ,t,P\right):=t_{0}-\psi \left(t^{⊤}\;{\bar{g}}^{c}\left(x,\theta ,P\right)\right).$$

Note that $g^{c}\left(x,\theta ,P_{\theta }\right)$ coincides with $g^{c}\left(x,\theta \right)$ defined at the beginning of this section. Throughout the paper, we also use the auxiliary function $n^{c}\left(x,\theta ,t,A,\tau \right)$ (when we need to work with the dependence on matrix A and vector τ) defined by

(31) $$n^{c}\left(x,\theta ,t,A,\tau \right):=t_{0}-\psi \left(t^{⊤}\;{\bar{h}}^{c}\left(x,\theta ,A,\tau \right)\right),\;$$

with ${\bar{h}}^{c}={\left(1_{R^{m}},{h^{c}}^{⊤}\right)}^{⊤}$. Note that

(32) $$\begin{array}{ccc} n^{c}\left(x,\theta ,t,A\left(\theta ,P\right),\tau \left(\theta ,P\right)\right) & = & t_{0}-\psi \left(t^{⊤}\;{\bar{h}}^{c}\left(x,\theta ,A\left(\theta ,P\right),\tau \left(\theta ,P\right)\right)\right)\\ & = & t_{0}-\psi \left(t^{⊤}\;{\bar{g}}^{c}\left(x,\theta ,P\right))=m^{c}(x,\theta ,t,P\right)\;. \end{array}$$

**Remark** **2. ** *For θ fixed, A(θ,P) and τ(θ,P) defined as solutions of (29) correspond to the estimators $A_{n}\left(\theta \right)$ and $\tau_{n}\left(\theta \right)$, which in fact are Z-estimators, whose *Ψ* function is specified in Section 2.4.1. In the proof of Proposition 1, in order to derive the influence functions of interest, we need $A(\theta ,{\tilde{P}}_{\varepsilon x})$ and $\tau (\theta ,{\tilde{P}}_{\varepsilon x})$ to be differentiable with respect to ε. Standard regularity conditions for Z-estimators can be used here as well. Continuous differentiability of the function (A,τ)↦Ψ(x,θ,A,τ), interchangeability of differentiation and integration, and nonsingularity of the Jacobian matrix at the solution, on the basis of the implicit function theorem, allow the differentiability with respect to ε of the above functionals.*

The following result is useful for computing the influence functions of the estimators.

**Lemma** **1.** *$t_{\theta_{0}}^{c}\left(P_{\theta_{0}}\right)={\left(\varphi^{\prime }\left(1\right),0^{⊤}\right)}^{⊤}=0\in R^{1+\ell }$, where l≥d.*

**Proposition** **1.** *(1) The influence function of the functional $t_{\theta }^{c}$ in $P_{\theta_{0}}$ is given by*

(33) $$\begin{array}{ccc} & & \mathrm{IF}\left(x;t_{\theta }^{c},P_{\theta_{0}}\right)=-{\left[\int \frac{\partial^{2}}{\partial^{2}t}n^{c}\left(y,\theta ,t_{\theta }^{c}\left(P_{\theta_{0}}\right),A\left(\theta ,P_{\theta_{0}}\right),\tau \left(\theta ,P_{\theta_{0}}\right)\right)\;dP_{\theta_{0}}\left(y\right)\right]}^{-1}\cdot \\ & & \cdot \left\{\sum_{i,j}\int \frac{\partial^{2}}{\partial a_{ij}\partial t}n^{c}\left(y,\theta ,t_{\theta }^{c}\left(P_{\theta_{0}}\right),A\left(\theta ,P_{\theta_{0}}\right),\tau \left(\theta ,P_{\theta_{0}}\right)\right)\;dP_{\theta_{0}}\left(y\right)\;\mathrm{IF}\left(x;a_{ij},P_{\theta_{0}}\right)+\right.\\ & & +\sum_{i}\int \frac{\partial^{2}}{\partial \tau_{i}\partial t}n^{c}\left(y,\theta ,t_{\theta }^{c}\left(P_{\theta_{0}}\right),A\left(\theta ,P_{\theta_{0}}\right),\tau \left(\theta ,P_{\theta_{0}}\right)\right)\;dP_{\theta_{0}}\left(y\right)\;\mathrm{IF}\left(x;\tau_{i},P_{\theta_{0}}\right)+\\ & & \left.+\frac{\partial }{\partial t}n^{c}\left(x,\theta ,t_{\theta }^{c}\left(P_{\theta_{0}}\right),A\left(\theta ,P_{\theta_{0}}\right),\tau \left(\theta ,P_{\theta_{0}}\right)\right)\right\}.\; \end{array}$$

*(2) The influence function of the functional $t_{\theta_{0}}^{c}$ in $P_{\theta_{0}}$ is*

(34) $$\begin{array}{ccc} \mathrm{IF}(x;t_{\theta_{0}}^{c},P_{\theta_{0}}) & = & -\varphi^{\prime \prime }\left(1\right){\left(0,h^{c}{\left(x,\theta_{0},A\left(\theta_{0},P_{\theta_{0}}\right),\tau \left(\theta_{0},P_{\theta_{0}}\right)\right)}^{⊤}\right)}^{⊤}\\ & = & -\varphi^{\prime \prime }\left(1\right){\left(0,g^{c}{\left(x,\theta_{0},P_{\theta_{0}}\right)}^{⊤}\right)}^{⊤}.\; \end{array}$$

For a given p.m. *P*, the statistical functional corresponding to the estimator ${\hat{\theta }}_{\varphi }^{c}$ is defined by

(35) $$T_{\varphi }^{c}\left(P\right):=\underset{\theta \in \Theta }{\arg\inf}\underset{t\in \Lambda_{\theta }^{c}\left(P\right)}{\sup}\;\int m^{c}\left(x,\theta ,t,P\right)\;dP\left(x\right).$$

**Proposition** **2.** *The influence function of the functional $T_{\varphi }^{c}$ in $P_{\theta_{0}}$ is given by*

(36) $$\begin{array}{ccc} \mathrm{IF}(x;T_{\varphi }^{c},P_{\theta_{0}}) & = & \;\;{\left\{{\left[\int \;\;\frac{\partial }{\partial \theta }g^{c}\left(y,\theta_{0},P_{\theta_{0}}\right)\;dP_{\theta_{0}}\left(y\right)\right]}^{⊤}\;\;\int \;\;\frac{\partial }{\partial \theta }\;g^{c}\left(y,\theta_{0},P_{\theta_{0}}\right)\;\;dP_{\theta_{0}}\left(y\right)\right\}}^{-1}\cdot \\ & & \cdot {\left[\int \frac{\partial }{\partial \theta }g^{c}\left(y,\theta_{0},P_{\theta_{0}}\right)\;dP_{\theta_{0}}\left(y\right)\right]}^{⊤}g^{c}\left(x,\theta_{0},P_{\theta_{0}}\right).\; \end{array}$$

The influence functions (34) and (36) are bounded with respect to *x*, irrespectively of the used divergence; therefore, the corresponding estimators are robust.

In the following, we establish an inequality for the self-standardized sensitivity of the estimator, which yields information regarding the possible choice of the tuning constant *c* appearing in the multivariate Huber function. According to [17] (p. 228), the self-standardized sensitivity is defined by

$$\gamma_{s}^{*}\left(T_{\varphi }^{c},P_{\theta_{0}}\right):=\underset{x}{\sup}{\left\{\mathrm{IF}{\left(x;T_{\varphi }^{c},P_{\theta_{0}}\right)}^{⊤}V{\left(T_{\varphi }^{c},P_{\theta_{0}}\right)}^{-1}\mathrm{IF}\left(x;T_{\varphi }^{c},P_{\theta_{0}}\right)\right\}}^{1/2}\;$$

where

$$V\left(T_{\varphi }^{c},P_{\theta_{0}}\right):=\int \mathrm{IF}\left(x;T_{\varphi }^{c},P_{\theta_{0}}\right)\mathrm{IF}{\left(x;T_{\varphi }^{c},P_{\theta_{0}}\right)}^{⊤}dP_{\theta_{0}}\left(x\right).$$

We show that the self-standardized sensitivity satisfies the inequality

$$\gamma_{s}^{*}\left(T_{\varphi }^{c},P_{\theta_{0}}\right)\leq c.\;$$

For the sake of simplicity, we denote $W_{0}:=\int \frac{\partial }{\partial \theta }g^{c}\left(y,\theta_{0},P_{\theta_{0}}\right)dP_{\theta_{0}}\left(y\right)$ and $V_{0}:=V\left(T_{\varphi }^{c},P_{\theta_{0}}\right).$ Using these notations, we have

$$\begin{array}{ccc} & & \mathrm{IF}\left(x;T_{\varphi }^{c},P_{\theta_{0}}\right)={\left\{W_{0}^{⊤}W_{0}\right\}}^{-1}W_{0}^{⊤}g^{c}\left(x,\theta_{0},P_{\theta_{0}}\right)\\ & & V_{0}=V\left(T_{\varphi }^{c},P_{\theta_{0}}\right)={\left\{W_{0}^{⊤}W_{0}\right\}}^{-1},\; \end{array}$$

taking into account that $\int g^{c}\left(x,\theta_{0},P_{\theta_{0}}\right)g^{c}{\left(x,\theta_{0},P_{\theta_{0}}\right)}^{⊤}dP_{\theta_{0}}\left(x\right)=I_{\ell }$, by construction of the function $g^{c}$. Then, the self-standardized sensitivity becomes

$$\begin{array}{ccc} \gamma_{s}^{*}\left(T_{\varphi }^{c},P_{\theta_{0}}\right) & = & \underset{x}{\sup}{\left\{g^{c}{\left(x,\theta_{0},P_{\theta_{0}}\right)}^{⊤}W_{0}{\left\{W_{0}^{⊤}W_{0}\right\}}^{-1}W_{0}^{⊤}g^{c}\left(x,\theta_{0},P_{\theta_{0}}\right)\right\}}^{1/2}\\ & \leq & \underset{x}{\sup}\{∥g^{c}\left(x,\theta_{0},P_{\theta_{0}}\right){∥}^{2}{\}}^{1/2}=\underset{x}{\sup}∥g^{c}\left(x,\theta_{0},P_{\theta_{0}}\right)∥\leq c\; \end{array}$$

The above inequality is obtained using the fact that $P:=W_{0}{\left\{W_{0}^{⊤}W_{0}\right\}}^{-1}W_{0}^{⊤}$ is an orthogonal projection matrix; namely, it satisfies two properties: it is symmetric $P^{⊤}=P$ and idempotent $P^{2}=P$. Since *P* is an orthogonal projection matrix, it cannot increase the Euclidean norm, so ${\left\{z^{⊤}Pz\right\}}^{1/2}=∥Pz∥\leq ∥z∥.$

So we obtain the inequality $\gamma_{s}^{*}\left(T_{\varphi }^{c},P_{\theta_{0}}\right)\leq c$. However, the bound imposed on the self-standardized sensitivity of an estimator cannot be chosen to be arbitrarily small. Indeed, $c\geq \sqrt{l}$, according to [17] (p. 228). The tuning constant *c* controls the degree of robustness of the estimator. In the numerical examples that we consider in Section 3, we choose a value close to the lower bound $\sqrt{l}$ to enhance the robustness of the estimator.

### 2.4. Asymptotic Properties

In this subsection, we establish some asymptotic properties of the estimators ${\hat{t}}_{\theta }^{c}$ and ${\hat{\theta }}_{\varphi }^{c}$. For this purpose, we first present asymptotic properties for the estimators $A_{n}\left(\theta \right)$ and $\tau_{n}\left(\theta \right)$ which are implicitly defined by the system (12).

#### 2.4.1. Asymptotic Properties of $A_{n}\left(\theta \right)$ and $\tau_{n}\left(\theta \right)$ for Fixed θ∈Θ

Note that $A_{n}\left(\theta \right)$ and $\tau_{n}\left(\theta \right)$ are Z-estimators of $A(\theta ,P_{\theta_{0}})$ and $\tau (\theta ,P_{\theta_{0}})$, respectively, since they are solutions of equation

(37) $$\int \Psi \left(x,\theta ,A_{n}\left(\theta \right),\tau_{n}\left(\theta \right)\right)\;dP_{n}\left(x\right)=0,\;$$

where $\Psi \left(x,\theta ,A,\tau \right):={\left(\Psi^{1}{\left(x,\theta ,A,\tau \right)}^{⊤},\mathrm{vec}\Psi^{2}{\left(x,\theta ,A,\tau \right)}^{⊤}\right)}^{⊤}$ with

(38) $$\begin{array}{ccc} \Psi^{1}\left(x,\theta ,A,\tau \right) & := & \int h^{c}\left(y,\theta ,A,\tau \right)\;dP_{\theta }\left(y\right) \end{array}$$

(39) $$\begin{array}{ccc} \Psi^{2}\left(x,\theta ,A,\tau \right) & := & h^{c}\left(x,\theta ,A,\tau \right)\;h^{c}{\left(x,\theta ,A,\tau \right)}^{⊤}-I_{\ell },\; \end{array}$$

“vec” being the operator that transforms the matrix into a vector by stacking all the columns of the matrix one underneath the other. In fact, $\Psi^{1}\left(x,\theta ,A,\tau \right)$ is a constant function with respect to *x*. The theoretical counterparts of $A_{n}\left(\theta \right)$ and $\tau_{n}\left(\theta \right)$ are $A(\theta ,P_{\theta_{0}})$ and $\tau (\theta ,P_{\theta_{0}})$ solutions of equation

(40) $$\int \Psi \left(x,\theta ,A\left(\theta ,P_{\theta_{0}}\right),\tau \left(\theta ,P_{\theta_{0}}\right)\right)\;dP_{\theta_{0}}\left(x\right)=0.\;$$

We refer to [18] (pp. 41–48) for definitions and asymptotic results concerning Z-estimators. Asymptotic results from the general theory of Z-estimators, as presented in [18], may be adapted in the present context.

In the following propositions, we consider a matrix *A* in its “vec” form, as defined above. This fact is necessary in order to apply some classical theoretical results, such as the Uniform Weak Law of Large Numbers (UWLLN) or results regarding Z-estimators. Therefore, the argument *A* of the functions $h^{c}$, $n^{c}$, and Ψ will in fact be vecA. For simplicity, we write *A* instead of vecA. The same is valid for $A_{n}\left(\theta \right)$ and $A(\theta ,P_{\theta_{0}})$.

**Assumption** **1. ** *(a) There exists a compact neighborhood $N_{A(\theta ,P_{\theta_{0}})}$ of $A(\theta ,P_{\theta_{0}})$ and a compact neighborhood $N_{\tau (\theta ,P_{\theta_{0}})}$ of $\tau (\theta ,P_{\theta_{0}})$ such that*

(41) $$\int \underset{A\in N_{A(\theta ,P_{\theta_{0}})},\tau \in N_{\tau (\theta ,P_{\theta_{0}})}}{\sup}∥\Psi \left(x,\theta ,A,\tau \right)∥\;dP_{\theta_{0}}\left(x\right)<\infty ;\;$$

*(b) For any positive ε, the following condition for the separability of a solution holds*

$$\begin{array}{c} \underset{(A,\tau )\in M}{\inf}∥\int \Psi \left(x,\theta ,A,\tau \right)\;dP_{\theta_{0}}\left(x\right)∥>0=∥\int \Psi \left(x,\theta ,A\left(\theta ,P_{\theta_{0}}\right),\tau \left(\theta ,P_{\theta_{0}}\right)\right)\;dP_{\theta_{0}}\left(x\right)∥ \end{array}$$

*where $M:=\left\{\left(A,\tau \right)\;s.t.\;∥\left(A,\tau \right)-(A\left(\theta ,P_{\theta_{0}}\right),\tau \left(\theta ,P_{\theta_{0}}\right))∥>\varepsilon \right\}$.*

**Proposition** **3.** *For each θ∈Θ, under Assumption 1, $A_{n}\left(\theta \right)$ converges in probability to $A(\theta ,P_{\theta_{0}})$ and $\tau_{n}\left(\theta \right)$ converges in probability to $\tau (\theta ,P_{\theta_{0}})$.*

**Assumption** **2. ** *(a) There exists a measurable function u(x) with the property $\int u{\left(x\right)}^{2}\;dP_{\theta_{0}}\left(x\right)<\infty$ and there exist $N_{A(\theta ,P_{\theta_{0}})}$ a neighborhood of $A(\theta ,P_{\theta_{0}})$ and $N_{\tau (\theta ,P_{\theta_{0}})}$ a neighborhood of $\tau (\theta ,P_{\theta_{0}})$ such that*

$$∥\Psi \left(x,\theta ,A_{1},\tau_{1}\right)-\Psi \left(x,\theta ,A_{2},\tau_{2}\right)∥\leq u\left(x\right)∥\left(A_{1},\tau_{1}\right)-\left(A_{2},\tau_{2}\right)∥,\;$$

*for any $\left(A_{1},\tau_{1}\right),\left(A_{2},\tau_{2}\right)\in N_{A(\theta ,P_{\theta_{0}})}\times N_{\tau (\theta ,P_{\theta_{0}})}$;*

*
(b) $\int ∥\Psi \left(x,\theta ,A\left(\theta ,P_{\theta_{0}}\right),\tau \left(\theta ,P_{\theta_{0}}\right)\right){∥}^{2}\;dP_{\theta_{0}}\left(x\right)<\infty ;$
*
*(c) the function $\left(A,\tau \right)\mapsto \int \Psi \left(x,\theta ,A,\tau \right)\;dP_{\theta_{0}}\left(x\right)$ is differentiable at $\left(A\left(\theta ,P_{\theta_{0}}\right),\tau \left(\theta ,P_{\theta_{0}}\right)\right)$ with nonsingular derivative matrix $V_{A\left(\theta ,P_{\theta_{0}}\right),\tau \left(\theta ,P_{\theta_{0}}\right)}$.*

**Proposition** **4.** *Under Assumptions 1 and 2,*

$$\sqrt{n}\left(\left(A_{n}\left(\theta \right),\tau_{n}\left(\theta \right)\right)-\left(A\left(\theta ,P_{\theta_{0}}\right),\tau \left(\theta ,P_{\theta_{0}}\right)\right)\right)$$

*is asymptotically normal with mean zero and covariance matrix*

$$\begin{array}{c} V_{A\left(\theta ,P_{\theta_{0}}\right),\tau \left(\theta ,P_{\theta_{0}}\right)}^{-1}\cdot \int \Psi \left(x,\theta ,A\left(\theta ,P_{\theta_{0}}\right),\tau \left(\theta ,P_{\theta_{0}}\right)\right)\Psi {\left(x,\theta ,A\left(\theta ,P_{\theta_{0}}\right),\tau \left(\theta ,P_{\theta_{0}}\right)\right)}^{⊤}\;dP_{\theta_{0}}\left(x\right)\cdot \\ {\left(V_{A\left(\theta ,P_{\theta_{0}}\right),\tau \left(\theta ,P_{\theta_{0}}\right)}^{-1}\right)}^{⊤}\; \end{array}$$

The proof is similar to that of Theorem 5.21 from [18] (p. 52).

#### 2.4.2. Asymptotic Properties of the Estimators

In order to prove the convergence in probability of ${\hat{t}}_{\theta }^{c}$ toward $t_{\theta }^{c}\left(P_{\theta_{0}}\right)$ for fixed θ∈Θ, the following assumptions are needed.

**Assumption** **3. ** There exist $n_{0}\in N^{*}$ and compact neighborhoods $N_{A(\theta ,P_{\theta_{0}})}$ of $A(\theta ,P_{\theta_{0}})$ and $N_{\tau (\theta ,P_{\theta_{0}})}$ of $\tau (\theta ,P_{\theta_{0}})$, such that $A_{n}\left(\theta \right)\in N_{A(\theta ,P_{\theta_{0}})}$ and $\tau_{n}\left(\theta \right)\in N_{\tau (\theta ,P_{\theta_{0}})}$, for all $n\geq n_{0}$.

**Assumption** **4. ** *(a) $t_{\theta }^{c}\left(P_{\theta_{0}}\right)=\underset{t\in \Lambda_{\theta }^{c}\left(P_{\theta_{0}}\right)}{\arg\sup}\;\int m^{c}\left(y,\theta ,t,P_{\theta_{0}}\right)\;dP_{\theta_{0}}\left(y\right)$ exists and is an interior point of $\Lambda_{\theta }^{c}\left(P_{\theta_{0}}\right)$;*

*(b) there exists a compact neighborhood $N_{t_{\theta }^{c}\left(P_{\theta_{0}}\right)}\subset \Lambda_{\theta }^{c}\left(P_{\theta_{0}}\right)$ such that $t_{\theta }^{c}\left(P_{\theta_{0}}\right)\in \mathrm{int}\left(N_{t_{\theta }^{c}\left(P_{\theta_{0}}\right)}\right)$, and there exist compact neighborhoods $N_{A(\theta ,P_{\theta_{0}})}$ of $A(\theta ,P_{\theta_{0}})$ and $N_{\tau (\theta ,P_{\theta_{0}})}$ of $\tau (\theta ,P_{\theta_{0}})$, such that*
$$\int \underset{t\in N_{t_{\theta }^{c}\left(P_{\theta_{0}}\right)},A\in N_{A(\theta ,P_{\theta_{0}})},\tau \in N_{\tau (\theta ,P_{\theta_{0}})}}{\sup}\left|n^{c}\left(x,\theta ,t,A,\tau \right)\right|\;\;dP_{\theta_{0}}\left(x\right)<\infty ;$$
*(c) the functions $1_{R^{m}},g_{1}^{c}\left(x,\theta ,P_{\theta_{0}}\right),\ldots ,g_{\ell }^{c}\left(x,\theta ,P_{\theta_{0}}\right)$ are linearly independent in the sense that*
$$P_{\theta_{0}}\left(\left\{x\in R^{m}\;s.t.\;t_{0}+\sum_{j=1}^{\ell }t_{j}g_{j}^{c}\left(x,\theta ,P_{\theta_{0}}\right)\neq 0\right\}\right)>0,\;for\;all\;t\in R^{1+\ell }\setminus \left\{0\right\};\;$$
*(d) there exists $N_{t_{\theta }^{c}\left(P_{\theta_{0}}\right)}$ a compact neighborhood of $t_{\theta }^{c}\left(P_{\theta_{0}}\right)$ and there exists a sequence $B_{n}=O_{P}\left(1\right)$, such that for all $t,t^{\prime }\in N_{t_{\theta }^{c}\left(P_{\theta_{0}}\right)}$ it holds*
$$\begin{array}{c} \left|\int n^{c}\left(x,\theta ,t,A_{n}\left(\theta \right),\tau_{n}\left(\theta \right)\right)\;dP_{\theta_{0}}\left(x\right)-\int n^{c}\left(x,\theta ,t^{\prime },A_{n}\left(\theta \right),\tau_{n}\left(\theta \right)\right)\;dP_{\theta_{0}}\left(x\right)\right|\leq B_{n}∥t-t^{\prime }∥.\; \end{array}$$

**Proposition** **5.** *Under Assumptions 1–4, it holds that*

*(1) ${\hat{t}}_{\theta }^{c}$ converges in probability to $t_{\theta }^{c}\left(P_{\theta_{0}}\right)$;*

*(2) ${\hat{D}}_{\varphi }\left(M_{\theta }^{c,n},P_{\theta_{0}}\right)$ converges in probability to $D_{\varphi }\left(M_{\theta }^{c},P_{\theta_{0}}\right)$.*

**Assumption** **5. ** *(a) *Θ* is compact;*

*(b) $h^{c}\left(X,\theta ,A,\tau \right)$ is continuous for each θ,A and τ with probability one;*

*(c) for each θ∈Θ, there exist $N_{t_{\theta }^{c}\left(P_{\theta_{0}}\right)}$, $N_{A(\theta ,P_{\theta_{0}})}$, $N_{\tau (\theta ,P_{\theta_{0}})}$ compact neighborhoods of $t_{\theta }^{c}\left(P_{\theta_{0}}\right)$, $A(\theta ,P_{\theta_{0}})$, and $\tau (\theta ,P_{\theta_{0}})$, respectively, such that the set C, defined as the closure of the set $\left\{\left(\theta ,t,A,\tau \right)\;s.t.\;\theta \in \Theta ,t\in N_{t_{\theta }^{c}\left(P_{\theta_{0}}\right)},A\in N_{A(\theta ,P_{\theta_{0}})},\tau \in N_{\tau (\theta ,P_{\theta_{0}})}\right\}$, is bounded and*

$$\int \underset{(\theta ,t,A,\tau )\in C}{\sup}\;\left|n^{c}\left(x,\theta ,t,A,\tau \right)\right|\;dP_{\theta_{0}}\left(x\right)<\infty ;$$

*(d) Let*

$$t_{n}^{*}\left(\theta \right):=\underset{t\in N_{t_{\theta }^{c}\left(P_{\theta_{0}}\right)}}{\arg\sup}\;\left|\int n^{c}\left(x,\theta ,t,A_{n}\left(\theta \right),\tau_{n}\left(\theta \right)\right)\;dP_{\theta_{0}}\left(x\right)-\int n^{c}\left(x,\theta ,t,A\left(\theta ,P_{\theta_{0}}\right),\tau \left(\theta ,P_{\theta_{0}}\right)\right)\;dP_{\theta_{0}}\left(x\right)\right|.$$

*There exists a sequence $B_{n}=O_{P}\left(1\right)$ such that for all $\theta ,\theta^{\prime }\in \Theta$ it holds*

$$\begin{array}{c} \left|\int n^{c}\left(x,\theta ,t_{n}^{*}\left(\theta \right),A_{n}\left(\theta \right),\tau_{n}\left(\theta \right)\right)\;dP_{\theta_{0}}\left(x\right)-\int n^{c}\left(x,\theta^{\prime },t_{n}^{*}\left(\theta^{\prime }\right),A_{n}\left(\theta^{\prime }\right),\tau_{n}\left(\theta^{\prime }\right)\right)\;dP_{\theta_{0}}\left(x\right)\right|\leq B_{n}∥\theta -\theta^{\prime }∥; \end{array}$$

*(e) the function $\theta \mapsto \int n^{c}\left(x,\theta ,t_{\theta }^{c}\left(P_{\theta_{0}}\right),A\left(\theta ,P_{\theta_{0}}\right),\tau \left(\theta ,P_{\theta_{0}}\right)\right)\;dP_{\theta_{0}}\left(x\right)$ is continuous on *Θ*;*

*(f)$\theta_{0}:=\underset{\theta \in \Theta }{\arg\inf}\int n^{c}\left(x,\theta ,t_{\theta }^{c}\left(P_{\theta_{0}}\right),A\left(\theta ,P_{\theta_{0}}\right),\tau \left(\theta ,P_{\theta_{0}}\right)\right)\;dP_{\theta_{0}}\left(x\right)$ exists, is unique and is an interior point of *Θ*.*

**Proposition** **6.** *Under Assumptions 1–5, it holds that*

*(1) $∥{\hat{t}}_{\theta }^{c}-t_{\theta }^{c}\left(P_{\theta_{0}}\right)∥\to 0$ in probability uniformly with respect to θ∈Θ;*

*(2) $∥{\hat{\theta }}_{\varphi }^{c}-\theta_{0}∥\to 0$ in probability.*

The following remark provides some insight into the assumptions underlying the theoretical results and explains the motivation behind them.

**Remark** **3. ** *Assumptions 1 and 2 are similar to those used in [18] to establish consistency and asymptotic normality of general Z-estimators.*

*Assumptions 4(a)–(c) and Assumptions 5(b)–(c) are motivated by those from [10], who introduced the original minimum empirical divergence estimators and studied their consistency. Similar conditions are standard in the literature on estimation under moment condition models. Furthermore, the boundedness of the proposed orthogonality functions allows some of these assumptions to be simplified compared with those required for the original estimators.*

*Using Assumption 4(b), we can apply UWLLN to obtain the uniform convergence in probability*

(42) $$\underset{t\in N_{t_{\theta }^{c}\left(P_{\theta_{0}}\right)},A\in N_{A(\theta ,P_{\theta_{0}})},\tau \in N_{\tau (\theta ,P_{\theta_{0}})}}{\sup}\left|\int n^{c}\left(x,\theta ,t,A,\tau \right)\;dP_{n}\left(x\right)-\int n^{c}\left(x,\theta ,t,A,\tau \right)\;dP_{\theta_{0}}\left(x\right)\right|\to 0,$$

*which, under Assumption 3, allows to prove the convergence in probability*

$$\underset{t\in N_{t_{\theta }^{c}\left(P_{\theta_{0}}\right)}}{\sup}\left|\int n^{c}\left(x,\theta ,t,A_{n}\left(\theta \right),\tau_{n}\left(\theta \right)\right)\;dP_{n}\left(x\right)-\int n^{c}\left(x,\theta ,t,A\left(\theta ,P_{\theta_{0}}\right),\tau \left(\theta ,P_{\theta_{0}}\right)\right)\;dP_{\theta_{0}}\left(x\right)\right|\to 0.\;$$

*Assumption 4(c) ensures the strict concavity of the function $t\in \Lambda_{\theta }^{c}\left(P_{\theta_{0}}\right)\mapsto \int m^{c}\left(y,\theta ,t,P_{\theta_{0}}\right)\;dP_{\theta_{0}}\left(y\right)$, on the convex set $\Lambda_{\theta }^{c}\left(P_{\theta_{0}}\right)$, which implies unicity of $t_{\theta }^{c}\left(P_{\theta_{0}}\right)$.*

*Assumptions 4(d) and 5(d) are motivated by Assumption 3A in Corollary 2.2 of [19], which provides sufficient conditions for upgrading pointwise convergence in probability to uniform convergence in probability over compact parameter spaces. Although somewhat technical, this hypothesis is consistent with the assumptions commonly adopted in the literature.*

## 3. Simulation Results

For illustrating the performance of the proposed minimum empirical divergence estimators, we consider, as an example, the moment condition model

$$\int_{R^{2}}g\left(x,\theta \right)\;dP_{0}\left(x\right),$$

where $g\left(x,\theta \right)={\left(x_{1},x_{2}-\theta \right)}^{⊤}$ and $P_{0}$ is the bi-variate Gaussian distribution with mean equals to ${\left(0,1\right)}^{⊤}$, both variances equal to 1 and the correlation coefficient equals to 0.6, with θ being the parameter of interest, and the true value being $\theta =\theta_{0}=1$. Such examples are common in survey sampling (see, e.g., [20,21]).

For the robust estimators, we used the truncated function $g^{c}\left(x,\theta \right)$ with c=1.5, which satisfies the condition $c\geq \sqrt{l}$, l=2. The choice of the constant *c* is in accordance with the results and comments presented at the end of Section 2.3. We chose a value close to the lower bound $\sqrt{l}$ to enhance the robustness of the estimator. We considered the estimator ${\hat{\theta }}_{\varphi }$ and the robust version ${\hat{\theta }}_{\varphi }^{c}$ for the cases of the K$L_{m}$, KL, $\chi^{2}$, and H divergences. As we mentioned in Remark 1 part (b), the choice of the K$L_{m}$ divergence corresponds to the empirical likelihood approach.

We computed the estimates on the basis of data generated from a slight deviation of the true model $P_{0}$, namely

(43) $$\left(1-\epsilon \right)P_{0}+\epsilon N,$$

where N is the bi-variate Gaussian distribution with mean equals to ${\left(-2,10\right)}^{⊤}$, with the same variance matrix as $P_{0}$ and ϵ is the contamination level. The simulation study was carried out by considering all combinations of three contamination levels ϵ∈{0.01,0.05,0.1} and three sample sizes n∈{50,100,200}. Consequently a total of nine simulation scenarios were examined. This design allows the assessment of the estimators’ performance across different degrees of contamination and sample sizes. For each considered divergence and corresponding estimation method, we used 1000 samples generated from the contaminated model (43) and computed the mean of the estimates, as well as their associated mean square errors in each case.

The algorithm for computing the estimates was obtained by adapting the algorithm from [8] (Appendix A.1, p. 3196) given for the robust exponential tilting estimator. Namely, for each iteration, the estimation of the parameter $\theta_{0}$, corresponding to the new orthogonality function in step iii, is computed using the minimum empirical divergence estimator defined in (21). We used the *fmincon()* procedure for optimization. All the computational aspects from Appendix A.2 in [8] were considered, but we have not included them here to save space.

**Figure 1:**
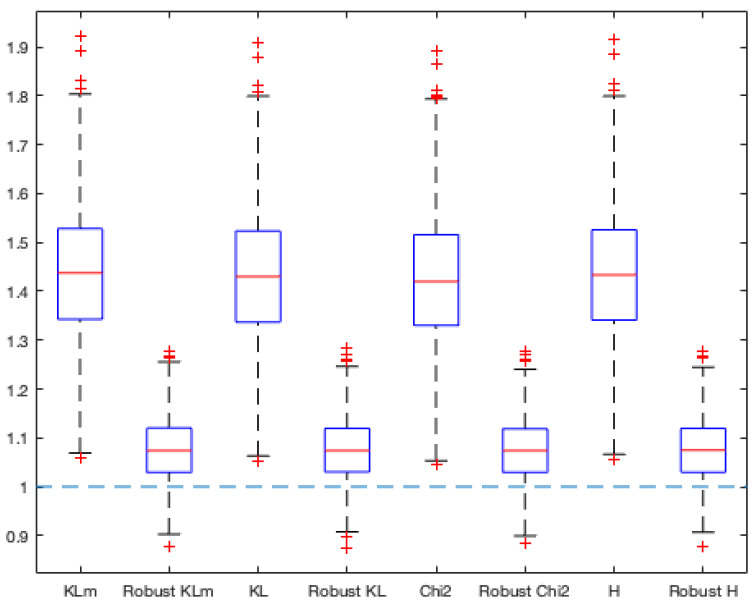
Boxplots for minimum empirical divergence estimations EL, KL, $\chi^{2}$, and H, respectively, for their robust versions Robust EL, Robust KL, Robust $\chi^{2}$, and Robust H, with ϵ=0.05 and n=200.

Table 1, Table 2 and Table 3 report the Monte Carlo means and mean squared errors (MSEs) of the proposed robust minimum empirical divergence estimators and of their classical counterparts for contaminated data, under the simulation scenarios described above. The results reveal a clear advantage of the robust procedures in the presence of contamination. While the original minimum empirical divergence estimations exhibit substantial departures from the true parameter value, the proposed robust estimations remain considerably closer to the true parameter. The robust estimators produce smaller MSE values than the corresponding classical estimators. This indicates that the Huber-type truncation effectively limits the impact of contaminated observations and prevents a small number of atypical observations from exerting excessive influence on the estimation procedure. For each fixed contamination level, the Monte Carlo results improve as the sample size increases. This behavior is expected, as larger sample sizes reduce the variability of the estimators, leading to smaller MSEs even in the presence of moderate contamination. Also, Figure 1 presents the boxplots for the classical and robust estimates on the basis of contaminated data. In this figure, EL, KL, $\chi^{2}$, and H represent ${\hat{\theta }}_{\varphi }$ and Robust EL, Robust KL, Robust $\chi^{2}$, Robust H represent ${\hat{\theta }}_{\varphi }^{c}$, using ${\mathrm{KL}}_{m}$, KL, $\chi^{2}$, and H divergences, respectively. Overall, the simulation results provide empirical support for the theoretical findings. The bounded influence robust minimum empirical divergence estimators retain stable performance under contamination and substantially outperform the minimum empirical divergence estimators in terms of estimation accuracy.

**Figure 2:**
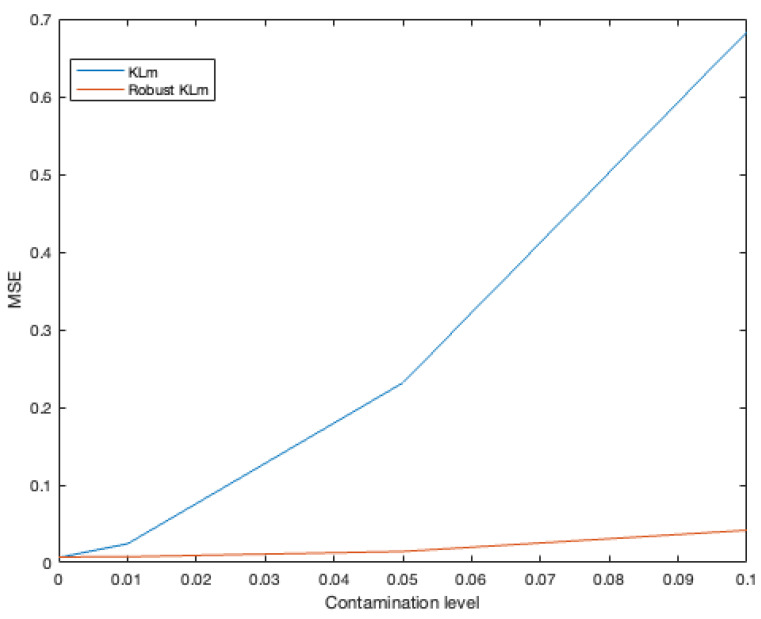
MSE comparison for the classical and robust minimum empirical divergence estimator, in the case of using K$L_{m}$ divergence, when c=1.5 and n=100.

**Figure 3:**
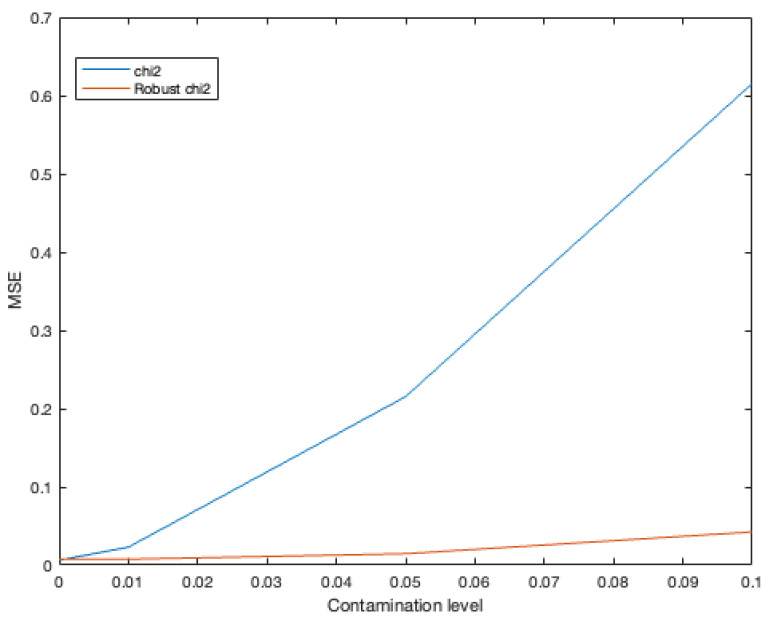
MSE comparison for the classical and robust minimum empirical divergence estimator, in the case of using χ2 divergence, when c=1.5 and n=100.

To assess the robustness–efficiency trade-off for the proposed estimators, the evolution of their mean squared error (MSE) with respect to different contamination levels was examined. Specifically, we considered ε∈{0,0.01,0.05,0.10}, where ε=0 corresponds to the uncontaminated setting and the larger values represent progressive departures from the assumed model. In the absence of contamination, the classical estimators achieve the lowest MSE, reflecting their optimal performance under the ideal model specification. However, their performance deteriorates rapidly as the contamination level increases, indicating their sensitivity to even small or moderate departures from the model. In contrast, the proposed robust estimators exhibit a small efficiency loss in the uncontaminated case but maintain a more stable MSE profile as contamination increases. Across the considered contamination scenarios, the robust estimators provide a more favorable compromise between efficiency and stability. Figure 2 and Figure 3 illustrate these results for the robust minimum empirical divergence estimators and their classical counterpart, in the cases of using the K$L_{m}$ and $\chi^{2}$ divergences, respectively. The proposed robust version of the minimum empirical divergence estimator based on the $\chi^{2}$ divergence is a novel contribution. As we mentioned in Remark 1 (c) concerning this estimator, the main advantage lies in the fact that the supremum involved in the dual representation admits an explicit expression, making the optimization step particularly simple. Moreover, while achieving an efficiency–robustness trade-off comparable to that of the KL and K$L_{m}$ divergence based estimators, it exhibits superior numerical stability, making it especially attractive from a computational perspective.

All these numerical results are consistent with the robustness properties targeted by the proposed methodology.

## 4. Conclusions

We introduced a class of robust estimators for moment condition models, representing robust alternatives to minimum empirical divergence estimators. They are constructed by using bounded truncated orthogonality functions and minimizing divergences in dual form, allowing to limit the impact of outliers or model deviations. The truncated functions are based on the multivariate Huber function, containing a shift vector and a scale matrix to manage outliers. The influence functions of the estimators are bounded; therefore, the estimators are robust, reducing sensitivity to outliers. The estimators are also consistent. These theoretical results together with the numerical results, based on Monte Carlo simulations, prove that extreme observations do not disproportionately affect the final estimates. The proposed methodology generates an entire family of robust estimators indexed by the choice of divergence. This unifies robust EL, robust ET, and many other estimators within a single coherent framework. Since minimum empirical divergence estimators have equivariance properties, the proposed framework opens the door to analyze equivariance properties for the proposed robust estimators and to study robust versions of minimum-risk equivariant estimators within this class and related divergence-based procedures. These issues will be considered in our future research.

## Figures and Tables

**Table 1:** Monte Carlo means and mean squared errors for minimum empirical divergence estimators and their robust versions in the case of contaminated data, when n=50 and c=1.5.

ϵ=0.01	K$L_{m}$	KL	$\chi^{2}$	H
${\hat{\theta }}_{\varphi }$	1.0923	1.0891	1.0857	1.0905
${\hat{\theta }}_{\varphi }^{c}$	1.0104	1.0105	1.0101	1.0100
MSE(${\hat{\theta }}_{\varphi }$)	0.0401	0.0387	0.0373	0.0394
MSE(${\hat{\theta }}_{\varphi }^{c}$)	0.0161	0.0163	0.0163	0.0162
ϵ=0.05	**K$L_{m}$**	**KL**	$\chi^{2}$	**H**
${\hat{\theta }}_{\varphi }$	1.4322	1.4222	1.4053	1.4290
${\hat{\theta }}_{\varphi }^{c}$	1.0808	1.0815	1.0821	1.0818
MSE(${\hat{\theta }}_{\varphi }$)	0.2662	0.2562	0.2414	0.2631
MSE(${\hat{\theta }}_{\varphi }^{c}$)	0.0249	0.0252	0.0255	0.0256
ϵ=0.10	**K$L_{m}$**	**KL**	$\chi^{2}$	**H**
${\hat{\theta }}_{\varphi }$	1.7707	1.7709	1.7347	1.7862
${\hat{\theta }}_{\varphi }^{c}$	1.1819	1.1814	1.1805	1.1802
MSE(${\hat{\theta }}_{\varphi }$)	0.7184	0.7136	0.6623	0.7380
MSE(${\hat{\theta }}_{\varphi }^{c}$)	0.0664	0.0650	0.0652	0.0640

**Table 2:** Monte Carlo means and mean squared errors for minimum empirical divergence estimators and their robust versions in the case of contaminated data, when n=100 and c=1.5.

ϵ=0.01	K$L_{m}$	KL	$\chi^{2}$	H
${\hat{\theta }}_{\varphi }$	1.0941	1.0919	1.0900	1.0927
${\hat{\theta }}_{\varphi }^{c}$	1.0096	1.0096	1.0099	1.0098
MSE(${\hat{\theta }}_{\varphi }$)	0.0242	0.0263	0.0230	0.0239
MSE(${\hat{\theta }}_{\varphi }^{c}$)	0.0076	0.0076	0.0076	0.0076
ϵ=0.05	**K$L_{m}$**	**KL**	$\chi^{2}$	**H**
${\hat{\theta }}_{\varphi }$	1.4389	1.4317	1.4212	1.4362
${\hat{\theta }}_{\varphi }^{c}$	1.0751	1.0750	1.0757	1.0753
MSE(${\hat{\theta }}_{\varphi }$)	0.2314	0.2245	0.2153	0.2287
MSE(${\hat{\theta }}_{\varphi }^{c}$)	0.0144	0.0144	0.0146	0.0145
ϵ=0.10	**K$L_{m}$**	**KL**	$\chi^{2}$	**H**
${\hat{\theta }}_{\varphi }$	1.7881	1.7786	1.7471	1.7925
${\hat{\theta }}_{\varphi }^{c}$	1.1662	1.1679	1.1673	1.1666
MSE(${\hat{\theta }}_{\varphi }$)	0.6832	0.6613	0.6153	0.6831
MSE(${\hat{\theta }}_{\varphi }^{c}$)	0.0417	0.0425	0.0424	0.0420

**Table 3:** Monte Carlo means and mean squared errors for minimum empirical divergence estimators and their robust versions in the case of contaminated data, when n=200 and c=1.5.

ϵ=0.01	K$L_{m}$	KL	$\chi^{2}$	H
${\hat{\theta }}_{\varphi }$	1.0948	1.0941	1.0933	1.0945
${\hat{\theta }}_{\varphi }^{c}$	1.0114	1.0115	1.0117	1.0115
MSE(${\hat{\theta }}_{\varphi }$)	0.0165	0.0163	0.0161	0.0164
MSE(${\hat{\theta }}_{\varphi }^{c}$)	0.0038	0.0038	0.0038	0.0038
ϵ=0.05	**K$L_{m}$**	**KL**	$\chi^{2}$	**H**
${\hat{\theta }}_{\varphi }$	1.4413	1.4349	1.4273	1.4382
${\hat{\theta }}_{\varphi }^{c}$	1.0749	1.0747	1.0749	1.0750
MSE(${\hat{\theta }}_{\varphi }$)	0.2148	0.2090	0.2025	0.2120
MSE(${\hat{\theta }}_{\varphi }^{c}$)	0.0098	0.0097	0.0097	0.0097
ϵ=0.10	**K$L_{m}$**	**KL**	$\chi^{2}$	**H**
${\hat{\theta }}_{\varphi }$	1.7912	1.7763	1.7501	1.7880
${\hat{\theta }}_{\varphi }^{c}$	1.1677	1.1698	1.1689	1.1690
MSE(${\hat{\theta }}_{\varphi }$)	0.6575	0.6305	0.5914	0.6489
MSE(${\hat{\theta }}_{\varphi }^{c}$)	0.0343	0.0354	0.0346	0.0348

## Data Availability

No new data were created or analyzed in this study.

## Abbreviations

The following abbreviations are used in this manuscript:

GMM	General Method of Moments
EL	Empirical Likelihood
CU	Continuous Updated
GEL	Generalized Empirical Likelihood
KL	Kullback–Leibler
K$L_{m}$	modified Kullback–Leibler
UWLLN	Uniform Weak Law of Large Numbers
p.m.	probability measure
a.c.	absolutely continuous
w.r.t.	with respect to

## Appendix A

**Proof** **of Lemma 1.** Indeed, for $P=P_{\theta_{0}}$, we have

$$g^{c}\left(x,\theta ,P_{\theta_{0}}\right)=h^{c}\left(x,\theta ,A\left(\theta ,P_{\theta_{0}}\right),\tau \left(\theta ,P_{\theta_{0}}\right)\right),$$

where $A(\theta ,P_{\theta_{0}})$ and $\tau (\theta ,P_{\theta_{0}})$ are solutions of the system (29), where *P* is replaced with $P_{\theta_{0}}$. Corresponding to the function $g^{c}\left(x,\theta ,P_{\theta_{0}}\right)$ (as a function of *x* and θ), we consider the set

(A1) $$M^{c}:=\underset{\theta \in \Theta }{⋃}M_{\theta }^{c},$$

where

(A2) $$M_{\theta }^{c}:=\left\{Q\in M\;s.t.\;\int dQ\left(x\right)=1\;\mathrm{and}\;\int g^{c}\left(x,\theta ,P_{\theta_{0}}\right)\;dQ\left(x\right)=0\right\}.$$

Since $g^{c}\left(x,\theta ,P_{\theta_{0}}\right)$ is bounded with respect to *x*, if we assume that $D_{\varphi }\left(M_{\theta }^{c},P_{\theta_{0}}\right)$ is finite, then on the basis of Corollary 1.2 from [16], the following dual representation of divergence holds

(A3) $$D_{\varphi }\left(M_{\theta }^{c},P_{\theta_{0}}\right)=\underset{t\in \Lambda_{\theta }^{c}\left(P_{\theta_{0}}\right)}{\sup}\int m^{c}\left(x,\theta ,t,P_{\theta_{0}}\right)\;dP_{\theta_{0}}\left(x\right).$$

Then $t_{\theta }^{c}\left(P_{\theta_{0}}\right)$, which is defined by (27) for $P=P_{\theta_{0}}$, represents in fact a solution of the optimization problem given by the dual representation (A3), namely,

(A4) $$t_{\theta }^{c}\left(P_{\theta_{0}}\right)=\underset{t\in \Lambda_{\theta }^{c}\left(P_{\theta_{0}}\right)}{\arg\sup}\;\int m^{c}\left(x,\theta ,t,P_{\theta_{0}}\right)\;dP_{\theta_{0}}\left(x\right).$$

For each θ∈Θ, the solution $t_{\theta }^{c}\left(P_{\theta_{0}}\right)$ in (A4) is unique. Similar arguments to those presented for the uniqueness of $t_{\theta }^{c,n}$ hold. Taking into account that $\inf_{\theta \in \Theta }D_{\varphi }\left(M_{\theta }^{c},P_{\theta_{0}}\right)=0$, the infimum being attained in $\theta =\theta_{0}$, and that

(A5) $$\theta \in \Theta \mapsto D_{\varphi }\left(M_{\theta }^{c},P_{\theta_{0}}\right)=\underset{t\in \Lambda_{\theta }^{c}\left(P_{\theta_{0}}\right)}{\sup}\int m^{c}\left(x,\theta ,t,P_{\theta_{0}}\right)\;dP_{\theta_{0}}\left(x\right)\geq 0,$$

for $\theta =\theta_{0}$, we have

(A6) $$\underset{t\in \Lambda_{\theta_{0}}^{c}\left(P_{\theta_{0}}\right)}{\sup}\int m^{c}\left(x,\theta_{0},t,P_{\theta_{0}}\right)\;dP_{\theta_{0}}\left(x\right)=0.$$

Note that, for $t^{*}={\left(\varphi^{\prime }\left(1\right),0^{⊤}\right)}^{⊤}=0\in R^{1+\ell }$, it holds that

$$m^{c}\left(x,\theta_{0},t^{*},P_{\theta_{0}}\right)=\varphi^{\prime }\left(1\right)-\varphi^{\prime }\left(1\right)\varphi^{\prime -1}\left(\varphi^{\prime }\left(1\right)\right)-\varphi \left(\varphi^{\prime -1}\left(\varphi^{\prime }\left(1\right)\right)\right)=-\varphi \left(1\right)=0.$$

By the uniqueness of $t_{\theta_{0}}^{c}\left(P_{\theta_{0}}\right)$ as a solution of the optimization problem (A4) for $\theta =\theta_{0}$, we get $t_{\theta_{0}}^{c}\left(P_{\theta_{0}}\right)=t^{*}={\left(\varphi^{\prime }\left(1\right),0^{⊤}\right)}^{⊤}=0\in R^{1+\ell }$. □

**Proof** **of Proposition 1.** (1) For the contaminated model ${\tilde{P}}_{\varepsilon x}=\left(1-\varepsilon \right)P_{\theta_{0}}+\varepsilon \delta_{x}$, using (27), $t_{\theta }^{c}\left({\tilde{P}}_{\varepsilon x}\right)$ is the solution of the equation

(A7) $$\int \frac{\partial }{\partial t}m^{c}\left(y,\theta ,t_{\theta }^{c}\left({\tilde{P}}_{\varepsilon x}\right),{\tilde{P}}_{\varepsilon x}\right)\;d{\tilde{P}}_{\varepsilon x}\left(y\right)=0,$$

which can also be written as

(A8) $$\int \frac{\partial }{\partial t}n^{c}\left(y,\theta ,t_{\theta }^{c}\left({\tilde{P}}_{\varepsilon x}\right),A\left(\theta ,{\tilde{P}}_{\varepsilon x}\right),\tau \left(\theta ,{\tilde{P}}_{\varepsilon x}\right)\right)\;d{\tilde{P}}_{\varepsilon x}\left(y\right)=0$$

on the basis of (32). It follows that

(A9) $$\begin{array}{cc} \left(1-\varepsilon \right)\int \frac{\partial }{\partial t}n^{c}(y,\theta ,t_{\theta }^{c}\left({\tilde{P}}_{\varepsilon x}\right),A\left(\theta ,{\tilde{P}}_{\varepsilon x}\right), & \tau \left(\theta ,{\tilde{P}}_{\varepsilon x}\right))\;dP_{\theta_{0}}\left(y\right)\\ & +\varepsilon \frac{\partial }{\partial t}n^{c}\left(x,\theta ,t_{\theta }^{c}\left({\tilde{P}}_{\varepsilon x}\right),A\left(\theta ,{\tilde{P}}_{\varepsilon x}\right),\tau \left(\theta ,{\tilde{P}}_{\varepsilon x}\right)\right)=0. \end{array}$$

Derivation with respect to ε in (A9) yields

(A10) $$\begin{array}{ccc} & & -\int \frac{\partial }{\partial t}n^{c}\left(y,\theta ,t_{\theta }^{c}\left(P_{\theta_{0}}\right),A\left(\theta ,P_{\theta_{0}}\right),\tau \left(\theta ,P_{\theta_{0}}\right)\right)\;dP_{\theta_{0}}\left(y\right)\\ & & +\int \frac{\partial^{2}}{\partial^{2}t}n^{c}\left(y,\theta ,t_{\theta }^{c}\left(P_{\theta_{0}}\right),A\left(\theta ,P_{\theta_{0}}\right),\tau \left(\theta ,P_{\theta_{0}}\right)\right)\;dP_{\theta_{0}}\left(y\right)\;\mathrm{IF}\left(x;t_{\theta }^{c},P_{\theta_{0}}\right)\\ & & +\sum_{i,j}\int \frac{\partial^{2}}{\partial a_{ij}\partial t}n^{c}\left(y,\theta ,t_{\theta }^{c}\left(P_{\theta_{0}}\right),A\left(\theta ,P_{\theta_{0}}\right),\tau \left(\theta ,P_{\theta_{0}}\right)\right)\;dP_{\theta_{0}}\left(y\right)\;\mathrm{IF}\left(x;a_{ij},P_{\theta_{0}}\right)\\ & & +\sum_{i}\int \frac{\partial^{2}}{\partial \tau_{i}\partial t}n^{c}\left(y,\theta ,t_{\theta }^{c}\left(P_{\theta_{0}}\right),A\left(\theta ,P_{\theta_{0}}\right),\tau \left(\theta ,P_{\theta_{0}}\right)\right)\;dP_{\theta_{0}}\left(y\right)\mathrm{IF}\left(x;\tau_{i},P_{\theta_{0}}\right)\\ & & +\frac{\partial }{\partial t}n^{c}\left(x,\theta ,t_{\theta }^{c}\left(P_{\theta_{0}}\right),A\left(\theta ,P_{\theta_{0}}\right),\tau \left(\theta ,P_{\theta_{0}}\right)\right)=0. \end{array}$$

Using (27) and (32), we obtain

$$\int \frac{\partial }{\partial t}n^{c}\left(y,\theta ,t_{\theta }^{c}\left(P_{\theta_{0}}\right),A\left(\theta ,P_{\theta_{0}}\right),\tau \left(\theta ,P_{\theta_{0}}\right)\right)dP_{\theta_{0}}\left(y\right)=0$$

which together with (A10) leads to

(A11) $$\begin{array}{ccc} & & \mathrm{IF}\left(x;t_{\theta }^{c},P_{\theta_{0}}\right)=-{\left[\int \frac{\partial^{2}}{\partial^{2}t}n^{c}\left(y,\theta ,t_{\theta }^{c}\left(P_{\theta_{0}}\right),A\left(\theta ,P_{\theta_{0}}\right),\tau \left(\theta ,P_{\theta_{0}}\right)\right)\;dP_{\theta_{0}}\left(y\right)\right]}^{-1}\\ & & \cdot \left\{\sum_{i,j}\int \frac{\partial^{2}}{\partial a_{ij}\partial t}n^{c}\left(y,\theta ,t_{\theta }^{c}\left(P_{\theta_{0}}\right),A\left(\theta ,P_{\theta_{0}}\right),\tau \left(\theta ,P_{\theta_{0}}\right)\right)dP_{\theta_{0}}\left(y\right)\;\mathrm{IF}\left(x;a_{ij},P_{\theta_{0}}\right)\right.\\ & & +\sum_{i}\int \frac{\partial^{2}}{\partial \tau_{i}\partial t}n^{c}\left(y,\theta ,t_{\theta }^{c}\left(P_{\theta_{0}}\right),A\left(\theta ,P_{\theta_{0}}\right),\tau \left(\theta ,P_{\theta_{0}}\right)\right)\;dP_{\theta_{0}}\left(y\right)\;\mathrm{IF}\left(x;\tau_{i},P_{\theta_{0}}\right)\\ & & \left.+\frac{\partial }{\partial t}n^{c}\left(x,\theta ,t_{\theta }^{c}\left(P_{\theta_{0}}\right),A\left(\theta ,P_{\theta_{0}}\right),\tau \left(\theta ,P_{\theta_{0}}\right)\right)\right\}. \end{array}$$

(2) Using definition (31) of function $n^{c}$ and the expression of function ψ given by (6), the derivative of $n^{c}$ with respect to *t* is

(A12) $$\begin{array}{cc} \frac{\partial }{\partial t}n^{c}\left(y,\theta ,t,A,\tau \right)=(1-\varphi^{\prime -1}(t^{⊤}\;{\bar{h}}^{c}(y,\theta , & A,\tau )),\\ & -h^{c}{\left(y,\theta ,A,\tau \right)}^{⊤}\varphi^{\prime -1}\left(t^{⊤}\;{\bar{h}}^{c}\left(y,\theta ,A,\tau \right)\right){)}^{⊤}. \end{array}$$

Then

(A13) $$\begin{array}{cc} \int \frac{\partial^{2}}{\partial^{2}t}n^{c}(y, & \theta ,t_{\theta }^{c}\left(P_{\theta_{0}}\right),A\left(\theta ,P_{\theta_{0}}\right),\tau \left(\theta ,P_{\theta_{0}}\right))\;dP_{\theta_{0}}\left(y\right)=\\ & -\int \frac{{\bar{h}}^{c}\left(y,\theta ,A\left(\theta ,P_{\theta_{0}}\right),\tau \left(\theta ,P_{\theta_{0}}\right)\right){\bar{h}}^{c}{\left(y,\theta ,A\left(\theta ,P_{\theta_{0}}\right),\tau \left(\theta ,P_{\theta_{0}}\right)\right)}^{⊤}}{\varphi^{\prime \prime }\left(\varphi^{\prime -1}\left(t_{\theta }^{c}{\left(P_{\theta_{0}}\right)}^{⊤}{\bar{h}}^{c}\left(y,\theta ,A\left(\theta ,P_{\theta_{0}}\right),\tau \left(\theta ,P_{\theta_{0}}\right)\right)\right)\right)}\;dP_{\theta_{0}}\left(y\right), \end{array}$$

using the fact that

$${\left(\varphi^{\prime -1}\right)}^{\prime }\left(u\right)=\frac{1}{\varphi^{\prime \prime }\left(\varphi^{\prime -1}\left(u\right)\right)}.$$

Using expression (A12) of $\frac{\partial }{\partial t}n^{c}$ and then differentiating with respect to $a_{ij}$, respectively, to $\tau_{i}$, we obtain that the sum of the last 3 terms in (A11) is

$$\begin{array}{ccc} & & \left(1-\varphi^{\prime -1}\left(t_{\theta }^{c}{\left(P_{\theta_{0}}\right)}^{⊤}{\bar{h}}^{c}\left(x,\theta ,A\left(\theta ,P_{\theta_{0}}\right),\tau \left(\theta ,P_{\theta_{0}}\right)\right)\right)\right.\\ & & -\sum_{i,j}\int \frac{t_{\theta }^{c}{\left(P_{\theta_{0}}\right)}^{⊤}\frac{\partial }{\partial a_{ij}}{\bar{h}}^{c}\left(y,\theta ,A\left(\theta ,P_{\theta_{0}}\right),\tau \left(\theta ,P_{\theta_{0}}\right)\right)}{\varphi^{\prime \prime }\left(\varphi^{\prime -1}\left(t_{\theta }^{c}{\left(P_{\theta_{0}}\right)}^{⊤}{\bar{h}}^{c}\left(y,\theta ,A\left(\theta ,P_{\theta_{0}}\right),\tau \left(\theta ,P_{\theta_{0}}\right)\right)\right)\right)}\;dP_{\theta_{0}}\left(y\right)\;\mathrm{IF}\left(x;a_{ij},P_{\theta_{0}}\right)\\ & & -\sum_{i}\int \frac{t_{\theta }^{c}{\left(P_{\theta_{0}}\right)}^{⊤}\frac{\partial }{\partial \tau_{i}}{\bar{h}}^{c}\left(y,\theta ,A\left(\theta ,P_{\theta_{0}}\right),\tau \left(\theta ,P_{\theta_{0}}\right)\right)}{\varphi^{\prime \prime }\left(\varphi^{\prime -1}\left(t_{\theta }^{c}{\left(P_{\theta_{0}}\right)}^{⊤}{\bar{h}}^{c}\left(y,\theta ,A\left(\theta ,P_{\theta_{0}}\right),\tau \left(\theta ,P_{\theta_{0}}\right)\right)\right)\right)}\;dP_{\theta_{0}}\left(y\right)\;\mathrm{IF}\left(x;\tau_{i},P_{\theta_{0}}\right),\\ & & \left(-h^{c}\left(x,\theta ,A\left(\theta ,P_{\theta_{0}}\right),\tau \left(\theta ,P_{\theta_{0}}\right)\right)\varphi^{\prime -1}\left(t_{\theta }^{c}{\left(P_{\theta_{0}}\right)}^{⊤}{\bar{h}}^{c}\left(x,\theta ,A\left(\theta ,P_{\theta_{0}}\right),\tau \left(\theta ,P_{\theta_{0}}\right)\right)\right)\right.\\ & & -\sum_{i,j}\left[\int \frac{h^{c}\left(y,\theta ,A\left(\theta ,P_{\theta_{0}}\right),\tau \left(\theta ,P_{\theta_{0}}\right)\right)t_{\theta }^{c}{\left(P_{\theta_{0}}\right)}^{⊤}\frac{\partial }{\partial a_{ij}}{\bar{h}}^{c}\left(y,\theta ,A\left(\theta ,P_{\theta_{0}}\right),\tau \left(\theta ,P_{\theta_{0}}\right)\right)}{\varphi^{\prime \prime }\left(\varphi^{\prime -1}\left(t_{\theta }^{c}{\left(P_{\theta_{0}}\right)}^{⊤}{\bar{h}}^{c}\left(y,\theta ,A\left(\theta ,P_{\theta_{0}}\right),\tau \left(\theta ,P_{\theta_{0}}\right)\right)\right)\right)}\;dP_{\theta_{0}}\left(y\right)\right.\\ & & +\int \varphi^{\prime -1}\left(t_{\theta }^{c}{\left(P_{\theta_{0}}\right)}^{⊤}{\bar{h}}^{c}\left(y,\theta ,A\left(\theta ,P_{\theta_{0}}\right),\tau \left(\theta ,P_{\theta_{0}}\right)\right)\right)\\ & & \left.\cdot \frac{\partial }{\partial a_{ij}}h^{c}\left(y,\theta ,A\left(\theta ,P_{\theta_{0}}\right),\tau \left(\theta ,P_{\theta_{0}}\right)\right)\;dP_{\theta_{0}}\left(y\right)\right]\;\mathrm{IF}\left(x;a_{ij},P_{\theta_{0}}\right) \end{array}$$

(A14) $$\begin{array}{ccc} & & -\sum_{i}\left[\int \frac{h^{c}\left(y,\theta ,A\left(\theta ,P_{\theta_{0}}\right),\tau \left(\theta ,P_{\theta_{0}}\right)\right)t_{\theta }^{c}{\left(P_{\theta_{0}}\right)}^{⊤}\frac{\partial }{\partial \tau_{i}}{\bar{h}}^{c}\left(y,\theta ,A\left(\theta ,P_{\theta_{0}}\right),\tau \left(\theta ,P_{\theta_{0}}\right)\right)}{\varphi^{\prime \prime }\left(\varphi^{\prime -1}\left(t_{\theta }^{c}{\left(P_{\theta_{0}}\right)}^{⊤}{\bar{h}}^{c}\left(y,\theta ,A\left(\theta ,P_{\theta_{0}}\right),\tau \left(\theta ,P_{\theta_{0}}\right)\right)\right)\right)}\;dP_{\theta_{0}}\left(y\right)\right.\\ & & +\int \varphi^{\prime -1}\left(t_{\theta }^{c}{\left(P_{\theta_{0}}\right)}^{⊤}{\bar{h}}^{c}\left(y,\theta ,A\left(\theta ,P_{\theta_{0}}\right),\tau \left(\theta ,P_{\theta_{0}}\right)\right)\right)\\ & & {\left.{\left.\left.\cdot \frac{\partial }{\partial \tau_{i}}h^{c}\left(y,\theta ,A\left(\theta ,P_{\theta_{0}}\right),\tau \left(\theta ,P_{\theta_{0}}\right)\right)\;dP_{\theta_{0}}\left(y\right)\right]\;\mathrm{IF}\left(x;\tau_{i},P_{\theta_{0}}\right)\right)}^{⊤}\right)}^{⊤}. \end{array}$$

We now consider the particular case $\theta =\theta_{0}$. Since $P_{\theta_{0}}\in M_{\theta_{0}}$, using Lemma 1, we have $t_{\theta_{0}}^{c}\left(P_{\theta_{0}}\right)={\left(\varphi^{\prime }\left(1\right),0_{\ell }^{⊤}\right)}^{⊤}=0_{\ell +1}$, also $\varphi^{\prime -1}\left(0\right)=1$. By replacing in (A13), we obtain the matrix

(A15) $$\begin{array}{c} -\int \frac{1}{\varphi^{\prime \prime }\left(1\right)}{\bar{h}}^{c}\left(y,\theta_{0},A\left(\theta_{0},P_{\theta_{0}}\right),\tau \left(\theta_{0},P_{\theta_{0}}\right)\right){\bar{h}}^{c}{\left(y,\theta_{0},A\left(\theta_{0},P_{\theta_{0}}\right),\tau \left(\theta_{0},P_{\theta_{0}}\right)\right)}^{⊤}\;dP_{\theta_{0}}\left(y\right)\\ =-\frac{1}{\varphi^{\prime \prime }\left(1\right)}I_{\ell +1} \end{array}$$

because

$$\int {\bar{h}}^{c}\left(y,\theta_{0},A\left(\theta_{0},P_{\theta_{0}}\right),\tau \left(\theta_{0},P_{\theta_{0}}\right)\right){\bar{h}}^{c}{\left(y,\theta_{0},A\left(\theta_{0},P_{\theta_{0}}\right),\tau \left(\theta_{0},P_{\theta_{0}}\right)\right)}^{⊤}\;dP_{\theta_{0}}\left(y\right)=I_{\ell +1}$$

taking into account relation (10) between functions $h^{c}$ and $g^{c}$ and definition (9) of the truncated function $g^{c}$. Also, in this particular case, the vector in (A14) becomes

$$\begin{array}{ccc} & & \left(0,\left(-h^{c}\left(x,\theta_{0},A\left(\theta_{0},P_{\theta_{0}}\right),\tau \left(\theta_{0},P_{\theta_{0}}\right)\right)-\right.\right.\\ & & -\sum_{i,j}\int \frac{\partial }{\partial a_{ij}}h^{c}\left(y,\theta_{0},A\left(\theta_{0},P_{\theta_{0}}\right),\tau \left(\theta_{0},P_{\theta_{0}}\right)\right)\;dP_{\theta_{0}}\left(y\right)\;\mathrm{IF}\left(x;a_{ij},P_{\theta_{0}}\right)\\ & & {\left.{\left.-\sum_{i}\int \frac{\partial }{\partial \tau_{i}}h^{c}\left(y,\theta_{0},A\left(\theta_{0},P_{\theta_{0}}\right),\tau \left(\theta_{0},P_{\theta_{0}}\right)\right)\;dP_{\theta_{0}}\left(y\right)\;\mathrm{IF}\left(x;\tau_{i},P_{\theta_{0}}\right)\right)}^{⊤}\right)}^{⊤}. \end{array}$$

Consequently, by replacing the above matrix and vector in (A11), we obtain

(A16) $$\begin{array}{ccc} & & \mathrm{IF}\left(x;t_{\theta_{0}}^{c},P_{\theta_{0}}\right)=-\varphi^{\prime \prime }\left(1\right)\left(0,h^{c}\left(x,\theta_{0},A\left(\theta_{0},P_{\theta_{0}}\right),\tau \left(\theta_{0},P_{\theta_{0}}\right)\right)\right.\\ & & +\sum_{i,j}\int \frac{\partial }{\partial a_{ij}}h^{c}\left(y,\theta_{0},A\left(\theta_{0},P_{\theta_{0}}\right),\tau \left(\theta_{0},P_{\theta_{0}}\right)\right)\;dP_{\theta_{0}}\left(y\right)\;\mathrm{IF}\left(x;a_{ij},P_{\theta_{0}}\right)\\ & & {\left.{\left.+\sum_{i}\int \frac{\partial }{\partial \tau_{i}}h^{c}\left(y,\theta_{0},A\left(\theta_{0},P_{\theta_{0}}\right),\tau \left(\theta_{0},P_{\theta_{0}}\right)\right)\;dP_{\theta_{0}}\left(y\right)\;\mathrm{IF}\left(x;\tau_{i},P_{\theta_{0}}\right)\right)}^{⊤}\right)}^{⊤}. \end{array}$$

For $\theta =\theta_{0}$ and the contaminated model ${\tilde{P}}_{\varepsilon x}=\left(1-\varepsilon \right)P_{\theta_{0}}+\varepsilon \delta_{x}$, it holds that

(A17) $$\int h^{c}\left(y,\theta_{0},A\left(\theta_{0},{\tilde{P}}_{\varepsilon x}\right),\tau \left(\theta_{0},{\tilde{P}}_{\varepsilon x}\right)\right)\;dP_{\theta_{0}}\left(y\right)=0.$$

Derivation with respect to ε in (A17) yields

(A18) $$\begin{array}{ccc} & & \sum_{i,j}\int \frac{\partial }{\partial a_{ij}}h^{c}\left(y,\theta_{0},A\left(\theta_{0},P_{\theta_{0}}\right),\tau \left(\theta_{0},P_{\theta_{0}}\right)\right)\;dP_{\theta_{0}}\left(y\right)\;\mathrm{IF}\left(x;a_{ij},P_{\theta_{0}}\right)\\ & & +\sum_{i}\int \frac{\partial }{\partial \tau_{i}}h^{c}\left(y,\theta_{0},A\left(\theta_{0},P_{\theta_{0}}\right),\tau \left(\theta_{0},P_{\theta_{0}}\right)\right)\;dP_{\theta_{0}}\left(y\right)\;\mathrm{IF}\left(x;\tau_{i},P_{\theta_{0}}\right)=0. \end{array}$$

By replacing (A18) in (A16), we obtain

(A19) $$\begin{array}{ccc} \mathrm{IF}(x;t_{\theta_{0}}^{c},P_{\theta_{0}}) & = & -\varphi^{\prime \prime }\left(1\right){\left(0,h^{c}{\left(x,\theta_{0},A\left(\theta_{0},P_{\theta_{0}}\right),\tau \left(\theta_{0},P_{\theta_{0}}\right)\right)}^{⊤}\right)}^{⊤}\\ & = & -\varphi^{\prime \prime }\left(1\right){\left(0,g^{c}{\left(x,\theta_{0},P_{\theta_{0}}\right)}^{⊤}\right)}^{⊤}. \end{array}$$

□

**Proof** **of Proposition 2.** The statistical functional corresponding to estimator ${\hat{\theta }}_{\varphi }^{c}$ is defined by

(A20) $$\begin{array}{ccc} T^{c}\left(P\right) & := & \underset{\theta \in \Theta }{\arg\inf}\underset{t\in \Lambda_{\theta }^{c}\left(P\right)}{\sup}\int m^{c}\left(y,\theta ,t,P\right)\;dP\left(y\right) \end{array}$$

(A21) $$\begin{array}{ccc} & = & \underset{\theta \in \Theta }{\arg\inf}\int m^{c}\left(y,\theta ,t_{\theta }^{c}\left(P\right),P\right)\;dP\left(y\right). \end{array}$$

We also use the notation $t^{c}\left(\theta ,P\right):=t_{\theta }^{c}\left(P\right)$. By the very definitions of $T^{c}\left(P\right)$ and $t^{c}\left(\theta ,P\right)$, they both obey

(A22) $$\begin{array}{ccc} & & \int \frac{\partial }{\partial t}m^{c}\left(y,\theta ,t^{c}\left(\theta ,P\right),P\right)\;dP\left(y\right)=0 \end{array}$$

(A23) $$\begin{array}{ccc} & & \int \frac{\partial }{\partial t}m^{c}\left(y,T^{c}\left(P\right),t^{c}\left(T^{c}\left(P\right),P\right),P\right)\;dP\left(y\right)=0 \end{array}$$

(A24) $$\begin{array}{ccc} & & \int \frac{\partial }{\partial \theta }{\left[m^{c}\left(y,\theta ,t^{c}\left(\theta ,P\right),P\right)\right]}_{\theta =T^{c}\left(P\right)}\;dP\left(y\right)=0. \end{array}$$

Using (30), we have

(A25) $$m^{c}\left(y,\theta ,t^{c}\left(\theta ,P\right),P\right)=t_{0}^{c}\left(\theta ,P\right)-\psi \left(t^{c}{\left(\theta ,P\right)}^{⊤}\;{\bar{h}}^{c}\left(y,\theta ,A\left(\theta ,P\right),\tau \left(\theta ,P\right)\right)\right),$$

and using the fact that $\psi^{\prime }\left(u\right)=\varphi^{\prime -1}\left(u\right)$, we obtain the derivative with respect to θ of $m^{c}$

(A26) $$\begin{array}{c} \frac{\partial }{\partial \theta }m^{c}\left(y,\theta ,t^{c}\left(\theta ,P\right),P\right)={\left[\frac{\partial }{\partial \theta }t^{c}\left(\theta ,P\right)\right]}^{⊤}\frac{\partial }{\partial t}m^{c}\left(y,\theta ,t^{c}\left(\theta ,P\right),P\right)\\ -\varphi^{\prime -1}\left(t^{c}{\left(\theta ,P\right)}^{⊤}\;{\bar{h}}^{c}\left(y,\theta ,A\left(\theta ,P\right),\tau \left(\theta ,P\right)\right)\right){\left[\frac{\partial }{\partial \theta }\left[{\bar{h}}^{c}\left(y,\theta ,A\left(\theta ,P\right),\tau \left(\theta ,P\right)\right)\right]\right]}^{⊤}t^{c}\left(\theta ,P\right). \end{array}$$

On the basis of (A23) and (A26), Equation (A24) becomes

(A27) $$\begin{array}{cc} \int \varphi^{\prime -1}(t^{c} & {\left(T^{c}\left(P\right),P\right)}^{⊤}\;{\bar{h}}^{c}\left(y,T^{c}\left(P\right),A\left(T^{c}\left(P\right),P\right),\tau \left(T^{c}\left(P\right),P\right)\right))\\ & \cdot {\left[\frac{\partial }{\partial \theta }\left[{\bar{h}}^{c}\left(y,T^{c}\left(P\right),A\left(T^{c}\left(P\right),P\right),\tau \left(T^{c}\left(P\right),P\right)\right)\right]\right]}^{⊤}t^{c}\left(T^{c}\left(P\right),P\right)\;dP\left(y\right)=0. \end{array}$$

On the basis of (A27), for the contaminated model ${\tilde{P}}_{\varepsilon x}$, $T^{c}\left({\tilde{P}}_{\varepsilon x}\right)$ is defined by

$$\begin{array}{cc} \int & \varphi^{\prime -1}\left(t^{c}{\left(T^{c}\left({\tilde{P}}_{\varepsilon x}\right),{\tilde{P}}_{\varepsilon x}\right)}^{⊤}\;{\bar{h}}^{c}\left(y,T^{c}\left({\tilde{P}}_{\varepsilon x}\right),A\left(T^{c}\left({\tilde{P}}_{\varepsilon x}\right),{\tilde{P}}_{\varepsilon x}\right),\tau \left(T^{c}\left({\tilde{P}}_{\varepsilon x}\right),{\tilde{P}}_{\varepsilon x}\right)\right)\right)\\ & \cdot {\left[\frac{\partial }{\partial \theta }\left[{\bar{h}}^{c}\left(y,T^{c}\left({\tilde{P}}_{\varepsilon x}\right),A\left(T^{c}\left({\tilde{P}}_{\varepsilon x}\right),{\tilde{P}}_{\varepsilon x}\right),\tau \left(T^{c}\left({\tilde{P}}_{\varepsilon x}\right),{\tilde{P}}_{\varepsilon x}\right)\right)\right]\right]}^{⊤}t^{c}\left(T^{c}\left({\tilde{P}}_{\varepsilon x}\right),{\tilde{P}}_{\varepsilon x}\right)\;d{\tilde{P}}_{\varepsilon x}\left(y\right)=0. \end{array}$$

Then,

$$\begin{array}{ccc} & & \left(1-\varepsilon \right)\int \varphi^{\prime -1}\left(t^{c}{\left(T^{c}\left({\tilde{P}}_{\varepsilon x}\right),{\tilde{P}}_{\varepsilon x}\right)}^{⊤}{\bar{h}}^{c}\left(y,T^{c}\left({\tilde{P}}_{\varepsilon x}\right),A\left(T^{c}\left({\tilde{P}}_{\varepsilon x}\right),{\tilde{P}}_{\varepsilon x}\right),\tau \left(T^{c}\left({\tilde{P}}_{\varepsilon x}\right),{\tilde{P}}_{\varepsilon x}\right)\right)\right)\\ & & \cdot {\left[\frac{\partial }{\partial \theta }\left[{\bar{h}}^{c}\left(y,T^{c}\left({\tilde{P}}_{\varepsilon x}\right),A\left(T^{c}\left({\tilde{P}}_{\varepsilon x}\right),{\tilde{P}}_{\varepsilon x}\right),\tau \left(T^{c}\left({\tilde{P}}_{\varepsilon x}\right),{\tilde{P}}_{\varepsilon x}\right)\right)\right]\right]}^{⊤}t^{c}\left(T^{c}\left({\tilde{P}}_{\varepsilon x}\right),{\tilde{P}}_{\varepsilon x}\right)\;dP_{\theta_{0}}\left(y\right)\\ & & +\varepsilon \varphi^{\prime -1}\left(t^{c}{\left(T^{c}\left({\tilde{P}}_{\varepsilon x}\right),{\tilde{P}}_{\varepsilon x}\right)}^{⊤}{\bar{h}}^{c}\left(x,T^{c}\left({\tilde{P}}_{\varepsilon x}\right),A\left(T^{c}\left({\tilde{P}}_{\varepsilon x}\right),{\tilde{P}}_{\varepsilon x}\right),\tau \left(T^{c}\left({\tilde{P}}_{\varepsilon x}\right),{\tilde{P}}_{\varepsilon x}\right)\right)\right)\\ & & \cdot {\left[\frac{\partial }{\partial \theta }\left[{\bar{h}}^{c}\left(x,T^{c}\left({\tilde{P}}_{\varepsilon x}\right),A\left(T^{c}\left({\tilde{P}}_{\varepsilon x}\right),{\tilde{P}}_{\varepsilon x}\right),\tau \left(T^{c}\left({\tilde{P}}_{\varepsilon x}\right),{\tilde{P}}_{\varepsilon x}\right)\right)\right]\right]}^{⊤}t^{c}\left(T^{c}\left({\tilde{P}}_{\varepsilon x}\right),{\tilde{P}}_{\varepsilon x}\right)=0. \end{array}$$

Derivation with respect to ε, taken in ε=0, yields

(A28) $$\begin{array}{ccc} & & -\int \varphi^{\prime -1}\left(t^{c}{\left(T^{c}\left(P_{\theta_{0}}\right),P_{\theta_{0}}\right)}^{⊤}\;{\bar{h}}^{c}\left(y,T^{c}\left(P_{\theta_{0}}\right),A\left(T^{c}\left(P_{\theta_{0}}\right),P_{\theta_{0}}\right),\tau \left(T^{c}\left(P_{\theta_{0}}\right),P_{\theta_{0}}\right)\right)\right)\\ & & \cdot {\left[\frac{\partial }{\partial \theta }\left[{\bar{h}}^{c}\left(y,T^{c}\left(P_{\theta_{0}}\right),A\left(T^{c}\left(P_{\theta_{0}}\right),P_{\theta_{0}}\right),\tau \left(T^{c}\left(P_{\theta_{0}}\right),P_{\theta_{0}}\right)\right)\right]\right]}^{⊤}t^{c}\left(T^{c}\left(P_{\theta_{0}}\right),P_{\theta_{0}}\right)\;dP_{\theta_{0}}\left(y\right)\\ & & +\int \frac{1}{\varphi^{\prime \prime }\left(\varphi^{\prime -1}\left(t^{c}{\left(T^{c}\left(P_{\theta_{0}}\right),P_{\theta_{0}}\right)}^{⊤}{\bar{h}}^{c}\left(y,T^{c}\left(P_{\theta_{0}}\right),A\left(T^{c}\left(P_{\theta_{0}}\right),P_{\theta_{0}}\right),\tau \left(T^{c}\left(P_{\theta_{0}}\right),P_{\theta_{0}}\right)\right)\right)\right)}\\ & & \cdot \frac{\partial }{\partial \varepsilon }{\left[t^{c}{\left(T^{c}\left({\tilde{P}}_{\varepsilon x}\right),{\tilde{P}}_{\varepsilon x}\right)}^{⊤}\;{\bar{h}}^{c}\left(y,T^{c}\left({\tilde{P}}_{\varepsilon x}\right),A\left(T^{c}\left({\tilde{P}}_{\varepsilon x}\right),{\tilde{P}}_{\varepsilon x}\right),\tau \left(T^{c}\left({\tilde{P}}_{\varepsilon x}\right),{\tilde{P}}_{\varepsilon x}\right)\right)\right]}_{\varepsilon =0}\\ & & \cdot {\left[\frac{\partial }{\partial \theta }\left[{\bar{h}}^{c}\left(y,T^{c}\left(P_{\theta_{0}}\right),A\left(T^{c}\left(P_{\theta_{0}}\right),P_{\theta_{0}}\right),\tau \left(T^{c}\left(P_{\theta_{0}}\right),P_{\theta_{0}}\right)\right)\right]\right]}^{⊤}t^{c}\left(T^{c}\left(P_{\theta_{0}}\right),P_{\theta_{0}}\right)\;dP_{\theta_{0}}\left(y\right)\\ & & +\int \varphi^{\prime -1}\left(t^{c}{\left(T^{c}\left(P_{\theta_{0}}\right),P_{\theta_{0}}\right)}^{⊤}{\bar{h}}^{c}\left(y,T^{c}\left(P_{\theta_{0}}\right),A\left(T^{c}\left(P_{\theta_{0}}\right),P_{\theta_{0}}\right),\tau \left(T^{c}\left(P_{\theta_{0}}\right),P_{\theta_{0}}\right)\right)\right)\\ & & \cdot \frac{\partial }{\partial \varepsilon }{\left[{\left[\frac{\partial }{\partial \theta }\left[{\bar{h}}^{c}\left(y,T^{c}\left({\tilde{P}}_{\varepsilon x}\right),A\left(T^{c}\left({\tilde{P}}_{\varepsilon x}\right),{\tilde{P}}_{\varepsilon x}\right),\tau \left(T^{c}\left({\tilde{P}}_{\varepsilon x}\right),{\tilde{P}}_{\varepsilon x}\right)\right)\right]\right]}^{⊤}\right]}_{\varepsilon =0}t^{c}\left(T^{c}\left(P_{\theta_{0}}\right),P_{\theta_{0}}\right)\;dP_{\theta_{0}}\left(y\right)\\ & & +\int \varphi^{\prime -1}\left(t^{c}{\left(T^{c}\left(P_{\theta_{0}}\right),P_{\theta_{0}}\right)}^{⊤}{\bar{h}}^{c}\left(y,T^{c}\left(P_{\theta_{0}}\right),A\left(T^{c}\left(P_{\theta_{0}}\right),P_{\theta_{0}}\right),\tau \left(T^{c}\left(P_{\theta_{0}}\right),P_{\theta_{0}}\right)\right)\right)\\ & & \cdot {\left[\frac{\partial }{\partial \theta }\left[{\bar{h}}^{c}\left(y,T^{c}\left(P_{\theta_{0}}\right),A\left(T^{c}\left(P_{\theta_{0}}\right),P_{\theta_{0}}\right),\tau \left(T^{c}\left(P_{\theta_{0}}\right),P_{\theta_{0}}\right)\right)\right]\right]}^{⊤}\;dP_{\theta_{0}}\left(y\right)\\ & & \cdot \left\{\frac{\partial }{\partial \theta }t^{c}\left(T^{c}\left(P_{\theta_{0}}\right),P_{\theta_{0}}\right)\mathrm{IF}\left(x;T^{c},P_{\theta_{0}}\right)+\mathrm{IF}\left(x;T_{T^{c}\left(P_{\theta_{0}}\right)},P_{\theta_{0}}\right)\right\}\\ & & +\varphi^{\prime -1}\left(t^{c}{\left(T^{c}\left(P_{\theta_{0}}\right),P_{\theta_{0}}\right)}^{⊤}{\bar{h}}^{c}\left(x,T^{c}\left(P_{\theta_{0}}\right),A\left(T^{c}\left(P_{\theta_{0}}\right),P_{\theta_{0}}\right),\tau \left(T^{c}\left(P_{\theta_{0}}\right),P_{\theta_{0}}\right)\right)\right)\cdot \\ & & {\left[\frac{\partial }{\partial \theta }\left[{\bar{h}}^{c}\left(x,T^{c}\left(P_{\theta_{0}}\right),A\left(T^{c}\left(P_{\theta_{0}}\right),P_{\theta_{0}}\right),\tau \left(T^{c}\left(P_{\theta_{0}}\right),P_{\theta_{0}}\right)\right)\right]\right]}^{⊤}t^{c}\left(T^{c}\left(P_{\theta_{0}}\right),P_{\theta_{0}}\right)=0. \end{array}$$

Since $T^{c}\left(P_{\theta_{0}}\right)=\theta_{0}$ and $t^{c}\left(T^{c}\left(P_{\theta_{0}}\right),P_{\theta_{0}}\right)=t_{\theta_{0}}^{c}\left(P_{\theta_{0}}\right)={\left(\varphi^{\prime }\left(1\right),0_{\ell }^{⊤}\right)}^{⊤}=0_{\ell +1}$, we have

(A29) $${\left[\frac{\partial }{\partial \theta }\left[{\bar{h}}^{c}\left(y,T^{c}\left(P_{\theta_{0}}\right),A\left(T^{c}\left(P_{\theta_{0}}\right),P_{\theta_{0}}\right),\tau \left(T^{c}\left(P_{\theta_{0}}\right),P_{\theta_{0}}\right)\right)\right]\right]}^{⊤}t^{c}\left(T^{c}\left(P_{\theta_{0}}\right),P_{\theta_{0}}\right)=0.$$

Consequently, the second, the third, and the last term in (A28) are all zero. Also, the first integral in (A28) is equal to zero according to the definition of $T^{c}\left(P_{\theta_{0}}\right)$, in analogy with (A27). Therefore, using the fact that $\varphi^{\prime -1}\left(0\right)=1,$ (A28) reduces to

(A30) $$\begin{array}{ccc} & & \int {\left[\frac{\partial }{\partial \theta }\left[{\bar{h}}^{c}\left(y,\theta_{0},A\left(\theta_{0},P_{\theta_{0}}\right),\tau \left(\theta_{0},P_{\theta_{0}}\right)\right)\right]\right]}^{⊤}\;dP_{\theta_{0}}\left(y\right)\cdot \\ & & \cdot \left\{\frac{\partial }{\partial \theta }t^{c}\left(\theta_{0},P_{\theta_{0}}\right)\mathrm{IF}\left(x;T^{c},P_{\theta_{0}}\right)+\mathrm{IF}\left(x;t_{\theta_{0}}^{c},P_{\theta_{0}}\right)\right\}=0. \end{array}$$

On the other hand, according to (A22), $t^{c}\left(\theta ,P\right)$ satisfies

$$\int \frac{\partial }{\partial t}m^{c}\left(y,\theta ,t^{c}\left(\theta ,P\right),P\right)\;dP\left(y\right)=0.$$

Derivation with respect to θ gives

(A31) $$\begin{array}{cc} \int \frac{\partial^{2}}{\partial^{2}t}m^{c}\left(y,\theta ,t^{c}\left(\theta ,P\right),P\right)\;dP\left(y\right)\;\frac{\partial }{\partial \theta }t^{c}\left(\theta ,P\right) & \\ & +\int \frac{\partial^{2}}{\partial \theta \partial t}m^{c}\left(y,\theta ,t^{c}\left(\theta ,P\right),P\right)\;dP\left(y\right)=0. \end{array}$$

Then,

(A32) $$\begin{array}{cc} \frac{\partial }{\partial \theta }t^{c}\left(\theta_{0},P_{\theta_{0}}\right)=-[\int \frac{\partial^{2}}{\partial^{2}t}m^{c}(y,\theta_{0},t^{c}(\theta_{0}, & P_{\theta_{0}}),P_{\theta_{0}})\;dP_{\theta_{0}}\left(y\right){]}^{-1}\\ & \cdot \int \frac{\partial^{2}}{\partial \theta \partial t}m^{c}\left(y,\theta_{0},t^{c}\left(\theta_{0},P_{\theta_{0}}\right),P_{\theta_{0}}\right)\;dP_{\theta_{0}}\left(y\right). \end{array}$$

According to (A13) and (A15), we get

(A33) $$\int \frac{\partial^{2}}{\partial^{2}t}m^{c}\left(y,\theta_{0},t^{c}\left(\theta_{0},P_{\theta_{0}}\right),P_{\theta_{0}}\right)\;dP_{\theta_{0}}\left(y\right)=-\frac{1}{\varphi^{\prime \prime }\left(1\right)}I_{\ell +1}.$$

On the other hand, using (A12), we obtain

$$\begin{array}{c} \frac{\partial^{2}}{\partial \theta \partial t}m^{c}\left(y,\theta_{0},t^{c}\left(\theta_{0},P_{\theta_{0}}\right),P_{\theta_{0}}\right)=\left(-t^{c}{\left(\theta_{0},P_{\theta_{0}}\right)}^{⊤}\frac{\partial }{\partial \theta }[{\bar{h}}^{c}\left(y,\theta_{0},A\left(\theta_{0},P_{\theta_{0}}\right),\tau \left(\theta_{0},P_{\theta_{0}}\right)\right]\right.\\ \cdot \frac{1}{\varphi^{\prime \prime }\left(\varphi^{\prime -1}\left(t^{c}{\left(\theta_{0},P_{\theta_{0}}\right)}^{⊤}{\bar{h}}^{c}\left(y,\theta_{0},A\left(\theta_{0},P_{\theta_{0}}\right),\tau \left(\theta_{0},P_{\theta_{0}}\right)\right)\right)\right)},\\ -h^{c}\left(y,\theta_{0},A\left(\theta_{0},P_{\theta_{0}}\right),\tau \left(\theta_{0},P_{\theta_{0}}\right)\right)t^{c}{\left(\theta_{0},P_{\theta_{0}}\right)}^{⊤}\\ \cdot \frac{\partial }{\partial \theta }[{\bar{h}}^{c}\left(y,\theta_{0},A\left(\theta_{0},P_{\theta_{0}}\right),\tau \left(\theta_{0},P_{\theta_{0}}\right)\right]\\ \cdot \frac{1}{\varphi^{\prime \prime }(\varphi^{\prime -1}(t^{c}{\left(\theta_{0},P_{\theta_{0}}\right)}^{⊤}{\bar{h}}^{c}\left(y,\theta_{0},A\left(\theta_{0},P_{\theta_{0}}\right),\tau \left(\theta_{0},P_{\theta_{0}}\right)\right)}\\ -\varphi^{\prime -1}(t^{c}{\left(\theta_{0},P_{\theta_{0}}\right)}^{⊤}{\bar{h}}^{c}\left(y,\theta_{0},A\left(\theta_{0},P_{\theta_{0}}\right),\tau \left(\theta_{0},P_{\theta_{0}}\right)\right)\\ \left.\cdot \frac{\partial }{\partial \theta }\left[h^{c}\left(y,\theta_{0},A\left(\theta_{0},P_{\theta_{0}}\right),\tau \left(\theta_{0},P_{\theta_{0}}\right)\right)\right]\right). \end{array}$$

Using the fact that $t^{c}\left(\theta_{0},P_{\theta_{0}}\right)={\left(\varphi^{\prime }\left(1\right),0_{\ell }^{⊤}\right)}^{⊤}$, we get

(A34) $$\begin{array}{cc} \int \frac{\partial^{2}}{\partial \theta \partial t}m^{c}\left(y,\theta_{0},t^{c}\left(\theta_{0},P_{\theta_{0}}\right),P_{\theta_{0}}\right) & dP_{\theta_{0}}\left(y\right)\\ =-(0,[\int \frac{\partial }{\partial \theta }[h^{c} & {{(y,\theta_{0},A\left(\theta_{0},P_{\theta_{0}}\right),\tau \left(\theta_{0},P_{\theta_{0}}\right))]\;dP_{\theta_{0}}\left(y\right)]}^{⊤})}^{⊤}\\ & =-\int \frac{\partial }{\partial \theta }{\bar{h}}^{c}\left(y,\theta_{0},A\left(\theta_{0},P_{\theta_{0}}\right),\tau \left(\theta_{0},P_{\theta_{0}}\right)\right)\;dP_{\theta_{0}}\left(y\right). \end{array}$$

From (A32), (A33), and (A34), we obtain

(A35) $$\frac{\partial }{\partial \theta }t^{c}\left(\theta_{0},P_{\theta_{0}}\right)=-\varphi^{\prime \prime }\left(1\right)\int \frac{\partial }{\partial \theta }{\bar{h}}^{c}\left(y,\theta_{0},A\left(\theta_{0},P_{\theta_{0}}\right),\tau \left(\theta_{0}\right),P_{\theta_{0}}\right)\;dP_{\theta_{0}}\left(y\right).$$

Then, from (A30), (A35), and (A19), we find

(A36) $$\begin{array}{ccc} \mathrm{IF}(x;T^{c},P_{\theta_{0}}) & = & -\frac{1}{\varphi^{\prime \prime }\left(1\right)}\left\{{\left[\int \frac{\partial }{\partial \theta }h^{c}\left(y,\theta_{0},A\left(\theta_{0},P_{\theta_{0}}\right),\tau \left(\theta_{0},P_{\theta_{0}}\right)\right)\;dP_{\theta_{0}}\left(y\right)\right]}^{⊤}\right.\\ & & {\left.\cdot \int \frac{\partial }{\partial \theta }h^{c}\left(y,\theta_{0},A\left(\theta_{0},P_{\theta_{0}}\right),\tau \left(\theta_{0},P_{\theta_{0}}\right)\right)\;dP_{\theta_{0}}\left(y\right)\right\}}^{-1}\\ & & \cdot {\left[\int \frac{\partial }{\partial \theta }h^{c}\left(y,\theta_{0},A\left(\theta_{0},P_{\theta_{0}}\right),\tau \left(\theta_{0},P_{\theta_{0}}\right)\right)\;dP_{\theta_{0}}\left(y\right)\right]}^{⊤}\\ & & \cdot \varphi^{\prime \prime }\left(1\right)h^{c}\left(x,\theta_{0},A\left(\theta_{0},P_{\theta_{0}}\right),\tau \left(\theta_{0},P_{\theta_{0}}\right)\right)). \end{array}$$

Consequently, given the relation between $h^{c}$ and $g^{c}$, we obtain

$$\begin{array}{ccc} \mathrm{IF}(x;T^{c},P_{\theta_{0}}) & = & -{\left\{{\left[\int \frac{\partial }{\partial \theta }g^{c}\left(y,\theta_{0},P_{\theta_{0}}\right)\;dP_{\theta_{0}}\left(y\right)\right]}^{⊤}\int \frac{\partial }{\partial \theta }g^{c}\left(y,\theta_{0},P_{\theta_{0}}\right)\;dP_{\theta_{0}}\left(y\right)\right\}}^{-1}\\ & & \cdot {\left[\int \frac{\partial }{\partial \theta }g^{c}\left(y,\theta_{0},P_{\theta_{0}}\right)\;dP_{\theta_{0}}\left(y\right)\right]}^{⊤}g^{c}\left(x,\theta_{0},P_{\theta_{0}}\right). \end{array}$$

□

**Proof** **of Proposition 3.** Since (A,τ)↦Ψ(x,A,τ) is continuous, by the uniform law of large numbers, Assumption 1 (a) implies

(A37) $$\underset{A\in N_{A(\theta ,P_{\theta_{0}})},\tau \in N_{\tau (\theta ,P_{\theta_{0}})}}{\sup}∥\int \Psi \left(x,\theta ,A,\tau \right)\;dP_{n}\left(x\right)-\int \Psi \left(x,\theta ,A,\tau \right)\;dP_{\theta_{0}}\left(x\right)∥\to 0$$

in probability. This result together with Assumption 1 (b) ensures the convergence in probability of estimators $A_{n}\left(\theta \right)$ and $\tau_{n}\left(\theta \right)$ toward $A(\theta ,P_{\theta_{0}})$ and $\tau (\theta ,P_{\theta_{0}})$, respectively. The arguments are the same as those from [18], Theorem 5.9, p.46. □

**Proof** **of Proposition 4.** (1) Assumption 4 (c) ensures the strict concavity of the function

$$t\in \Lambda_{\theta }^{c}\left(P_{\theta_{0}}\right)\mapsto \int m^{c}\left(y,\theta ,t,P_{\theta_{0}}\right)\;dP_{\theta_{0}}\left(y\right)$$

on the convex set $\Lambda_{\theta }^{c}\left(P_{\theta_{0}}\right)$, which implies that $t_{\theta }^{c}\left(P_{\theta_{0}}\right)$ is unique. Using Assumption 4 (b) and the continuity of function $n^{c}\left(x,\theta ,t,A,\tau \right)$ with respect to t,A and τ, by UWLLN, we get

(A38) $$M_{n}:=\underset{t\in N_{t_{\theta }^{c}\left(P_{\theta_{0}}\right)},A\in N_{A(\theta ,P_{\theta_{0}})},\tau \in N_{\tau (\theta ,P_{\theta_{0}})}}{\sup}\left|\int n^{c}\left(x,\theta ,t,A,\tau \right)\;dP_{n}\left(x\right)-\int n^{c}\left(x,\theta ,t,A,\tau \right)\;dP_{\theta_{0}}\left(x\right)\right|\to 0$$

in probability. The following inequality holds

(A39) $$\begin{array}{c} \underset{t\in N_{t_{\theta }^{c}\left(P_{\theta_{0}}\right)}}{\sup}\left|\int n^{c}\left(x,\theta ,t,A_{n}\left(\theta \right),\tau_{n}\left(\theta \right)\right)\;dP_{n}\left(x\right)-\int n^{c}\left(x,\theta ,t,A\left(\theta ,P_{\theta_{0}}\right),\tau \left(\theta ,P_{\theta_{0}}\right)\right)\;dP_{\theta_{0}}\left(x\right)\right|\leq \\ \underset{t\in N_{t_{\theta }^{c}\left(P_{\theta_{0}}\right)}}{\sup}\left|\int n^{c}\left(x,\theta ,t,A_{n}\left(\theta \right),\tau_{n}\left(\theta \right)\right)\;dP_{n}\left(x\right)-\int n^{c}\left(x,\theta ,t,A_{n}\left(\theta \right),\tau_{n}\left(\theta \right)\right)\;dP_{\theta_{0}}\left(x\right)\right|\\ +\underset{t\in N_{t_{\theta }^{c}\left(P_{\theta_{0}}\right)}}{\sup}\left|\int n^{c}\left(x,\theta ,t,A_{n}\left(\theta \right),\tau_{n}\left(\theta \right)\right)\;dP_{\theta_{0}}\left(x\right)-\int n^{c}\left(x,\theta ,t,A\left(\theta ,P_{\theta_{0}}\right),\tau \left(\theta ,P_{\theta_{0}}\right)\right)\;dP_{\theta_{0}}\left(x\right)\right|. \end{array}$$

The first term on the right-hand side of the inequality (A39) tends to 0 in probability on the basis of the result (42) and using Assumption 3. The second term on the right-hand side of (A39) also tends to 0 in probability. Using the dominated convergence theorem, under Assumption 4 (b), we get the pointwise convergence for each *t*. Then, according to Corollary 2.1 from [19], using the pointwise convergence together with Assumption 4 (d), we obtain the uniform convergence

(A40) $$\underset{t\in N_{t_{\theta }^{c}\left(P_{\theta_{0}}\right)}}{\sup}\left|\int n^{c}\left(x,\theta ,t,A_{n}\left(\theta \right),\tau_{n}\left(\theta \right)\right)\;dP_{\theta_{0}}\left(x\right)-\int n^{c}\left(x,\theta ,t,A\left(\theta ,P_{\theta_{0}}\right),\tau \left(\theta ,P_{\theta_{0}}\right)\right)\;dP_{\theta_{0}}\left(x\right)\right|=o_{P}\left(1\right).\;$$

Consequently,

(A41) $$\underset{t\in N_{t_{\theta }^{c}\left(P_{\theta_{0}}\right)}}{\sup}\left|\int n^{c}\left(x,\theta ,t,A_{n}\left(\theta \right),\tau_{n}\left(\theta \right)\right)\;dP_{n}\left(x\right)-\int n^{c}\left(x,\theta ,t,A\left(\theta ,P_{\theta_{0}}\right),\tau \left(\theta ,P_{\theta_{0}}\right)\right)\;dP_{\theta_{0}}\left(x\right)\right|\to 0\;$$

in probability. Using (A41), the fact that $t_{\theta }^{c}\left(P_{\theta_{0}}\right)$ is unique and belongs to $\mathrm{int}(N_{t_{\theta }^{c}\left(P_{\theta_{0}}\right)})$, as well as the strict concavity of the function

$$t\mapsto \int n^{c}\left(y,\theta ,t,A\left(\theta ,P_{\theta_{0}}\right),\tau \left(\theta ,P_{\theta_{0}}\right)\right)\;dP_{\theta_{0}}\left(y\right),$$

on the basis of Theorem 5.7 in [18], we conclude that any value

(A42) $$\bar{t}:=\underset{t\in N_{t_{\theta }^{c}\left(P_{\theta_{0}}\right)}}{\arg\sup}\int n^{c}\left(x,\theta ,t,A_{n}\left(\theta \right),\tau_{n}\left(\theta \right)\right)\;dP_{n}\left(x\right)$$

converges in probability to $t_{\theta }^{c}\left(P_{\theta_{0}}\right)$. We show that ${\hat{t}}_{\theta }^{c}$ belongs to $\mathrm{int}(N_{t_{\theta }^{c}\left(P_{\theta_{0}}\right)})$ with probability one as n→∞, and consequently, it converges to $t_{\theta }^{c}\left(P_{\theta_{0}}\right)$. Since for *n* sufficiently large any $\bar{t}\in \mathrm{int}\left(N_{t_{\theta }^{c}\left(P_{\theta_{0}}\right)}\right)$, the concavity of the function $t\mapsto \int n^{c}\left(x,\theta ,t,A_{n}\left(\theta \right),\tau_{n}\left(\theta \right)\right)\;dP_{n}\left(x\right)$ ensures that no other point *t* in the complement of $\mathrm{int}(N_{t_{\theta }^{c}\left(P_{\theta_{0}}\right)})$ can maximize $\int n^{c}(x,\theta ,t,A_{n}\left(\theta \right)$, $\tau_{n}\left(\theta \right))\;dP_{n}\left(x\right)$ over $t\in R^{1+\ell }$; hence, ${\hat{t}}_{\theta }^{c}$ belongs to $\mathrm{int}(N_{t_{\theta }^{c}\left(P_{\theta_{0}}\right)})$. (2) It holds

$$\begin{array}{ccc} {\hat{D}}_{\varphi }\left(M_{\theta }^{c,n},P_{\theta_{0}}\right)-D_{\varphi }\left(M_{\theta }^{c},P_{\theta_{0}}\right) & = & \int n^{c}\left(x,\theta ,{\hat{t}}_{\theta }^{c},A_{n}\left(\theta \right),\tau_{n}\left(\theta \right)\right)\;dP_{n}\left(x\right)-\\ & & -\int n^{c}\left(x,\theta ,t_{\theta }^{c}\left(P_{\theta_{0}}\right),A\left(\theta ,P_{\theta_{0}}\right),\tau \left(\theta ,P_{\theta_{0}}\right)\right)\;dP_{\theta_{0}}\left(x\right). \end{array}$$

Note that

(A43) $$\begin{array}{ccc} & & \int n^{c}\left(x,\theta ,t_{\theta }^{c}\left(P_{\theta_{0}}\right),A_{n}\left(\theta \right),\tau_{n}\left(\theta \right)\right)\;dP_{n}\left(x\right)-\int n^{c}\left(x,\theta ,t_{\theta }^{c}\left(P_{\theta_{0}}\right),A\left(\theta ,P_{\theta_{0}}\right),\tau \left(\theta ,P_{\theta_{0}}\right)\right)\;dP_{\theta_{0}}\left(x\right)\\ & & \leq {\hat{D}}_{\varphi }\left(M_{\theta }^{c,n},P_{\theta_{0}}\right)-D_{\varphi }\left(M_{\theta },P_{\theta_{0}}\right)\leq \\ & & \int n^{c}\left(x,\theta ,{\hat{t}}_{\theta }^{c},A_{n}\left(\theta \right),\tau_{n}\left(\theta \right)\right)\;dP_{n}\left(x\right)-\int n^{c}\left(x,\theta ,{\hat{t}}_{\theta }^{c},A\left(\theta ,P_{\theta_{0}}\right),\tau \left(\theta ,P_{\theta_{0}}\right)\right)\;dP_{\theta_{0}}\left(x\right). \end{array}$$

Both the right-hand side and the left-hand side in the above display tend to 0 in probability using (A41). Hence ${\hat{D}}_{\varphi }\left(M_{\theta }^{c,n},P_{\theta_{0}}\right)$ converges to $D_{\varphi }\left(M_{\theta }^{c},P_{\theta_{0}}\right)$ in probability. □

**Proof** **of Proposition 5.** (1) Assumption 4 (c) implies that the function

(A44) $$t\in \Lambda_{\theta }^{c}\left(P_{\theta_{0}}\right)\to \int n^{c}\left(x,\theta ,t,A\left(\theta ,P_{\theta_{0}}\right),\tau \left(\theta ,P_{\theta_{0}}\right)\right)\;dP_{\theta_{0}}\left(x\right)$$

is strictly concave for any θ∈Θ, which implies that $t_{\theta }^{c}\left(P_{\theta_{0}}\right)$ is unique for any θ∈Θ. By Assumption 5 (b), $n^{c}\left(x,\theta ,t,A,\tau \right)$ is continuous in θ,t,A,τ. Using also Assumption 5 (c), by applying UWLLN, we obtain the uniform convergence in probability

(A45) $$\underset{(\theta ,t,A,\tau )\in C}{\sup}\left|\int n^{c}\left(x,\theta ,t,A,\tau \right)\;dP_{n}\left(x\right)-\int n^{c}\left(x,\theta ,t,A,\tau \right)\;dP_{\theta_{0}}\left(x\right)\right|\to 0$$

over the compact set *C*, the closure of the set

(A46) $$C:=\left\{\left(\theta ,t,A,\tau \right)\;s.t.\;\theta \in \Theta ,t\in N_{t_{\theta }^{c}\left(P_{\theta_{0}}\right)},A\in N_{A(\theta ,P_{\theta_{0}})},\tau \in N_{\tau (\theta ,P_{\theta_{0}})}\right\}.$$

We prove the uniform convergence in probability

(A47) $$\underset{\theta \in \Theta }{\sup}∥{\hat{t}}_{\theta }^{c}-t_{\theta }^{c}\left(P_{\theta_{0}}\right)∥\to 0\;\mathrm{in}\;\mathrm{probability}$$

in two steps.

Step 1. Let η>0. We show that $P_{\theta_{0}}(\sup_{\theta \in \Theta }∥{\bar{t}}_{\theta }^{c}-t_{\theta }^{c}\left(P_{\theta_{0}}\right)∥\geq \eta )\to 0$ for any
  (A48) $${\bar{t}}_{\theta }^{c}:=\underset{t\in N_{t_{\theta }^{c}\left(P_{\theta_{0}}\right)}}{\arg\sup}\int n^{c}\left(x,\theta ,t,A_{n}\left(\theta \right),\tau_{n}\left(\theta \right)\right)\;dP_{n}\left(x\right).$$
Step 2. We show that ${\hat{t}}_{\theta }^{c}$ belongs to $\mathrm{int}(N_{t_{\theta }^{c}\left(P_{\theta_{0}}\right)})$ with probability one, as n→∞. Let η>0 be such that $\sup_{\theta \in \Theta }∥{\bar{t}}_{\theta }^{c}-t_{\theta }^{c}\left(P_{\theta_{0}}\right)∥\geq \eta$. Since Θ is compact, by continuity, there exists $\bar{\theta }\in \Theta$, such that $\sup_{\theta \in \Theta }∥{\bar{t}}_{\theta }^{c}-t_{\theta }^{c}\left(P_{\theta_{0}}\right)∥=∥{\bar{t}}_{\bar{\theta }}^{c}-t_{\bar{\theta }}^{c}\left(P_{\theta_{0}}\right)∥\geq \eta .$ Hence, there exists ε>0 such that

$$\begin{array}{cc} \int n^{c}\left(x,\bar{\theta },t_{\bar{\theta }}^{c}\left(P_{\theta_{0}}\right),A\left(\bar{\theta },P_{\theta_{0}}\right),\tau \left(\bar{\theta },P_{\theta_{0}}\right)\right)\;d & P_{\theta_{0}}\left(x\right)\\ & -\int n^{c}\left(x,\bar{\theta },{\bar{t}}_{\bar{\theta }}^{c},A\left(\bar{\theta },P_{\theta_{0}}\right),\tau \left(\bar{\theta },P_{\theta_{0}}\right)\right)\;dP_{\theta_{0}}\left(x\right)>\varepsilon . \end{array}$$

Hence,

(A49) $$\begin{array}{ccc} P_{\theta_{0}}\left(\underset{\theta \in \Theta }{\sup}∥{\bar{t}}_{\theta }^{c}-t_{\theta }^{c}\left(P_{\theta_{0}}\right)∥\geq \eta \right) & \leq & P_{\theta_{0}}\left(\int n^{c}\left(x,\bar{\theta },t_{\bar{\theta }}^{c}\left(P_{\theta_{0}}\right),A\left(\bar{\theta },P_{\theta_{0}}\right),\tau \left(\bar{\theta },P_{\theta_{0}}\right)\right)\;dP_{\theta_{0}}\left(x\right)-\right.\\ & & \left.\int n^{c}\left(x,\bar{\theta },{\bar{t}}_{\bar{\theta }}^{c},A\left(\bar{\theta },P_{\theta_{0}}\right),\tau \left(\bar{\theta },P_{\theta_{0}}\right)\right)\;dP_{\theta_{0}}\left(x\right)>\varepsilon \right). \end{array}$$

On the other hand, using (A41) and (A40), we can write

(A50) $$\begin{array}{c} \int n^{c}\left(x,\bar{\theta },t_{\bar{\theta }}^{c}\left(P_{\theta_{0}}\right),A\left(\bar{\theta },P_{\theta_{0}}\right),\tau \left(\bar{\theta },P_{\theta_{0}}\right)\right)\;dP_{\theta_{0}}\left(x\right)-\int n^{c}\left(x,\bar{\theta },{\bar{t}}_{\bar{\theta }}^{c},A\left(\bar{\theta },P_{\theta_{0}}\right),\tau \left(\bar{\theta },P_{\theta_{0}}\right)\right)\;dP_{\theta_{0}}\left(x\right)=\\ \int n^{c}\left(x,\bar{\theta },t_{\bar{\theta }}^{c}\left(P_{\theta_{0}}\right),A_{n}\left(\bar{\theta }\right),\tau_{n}\left(\bar{\theta }\right)\right)\;dP_{n}\left(x\right)-\int n^{c}\left(x,\bar{\theta },{\bar{t}}_{\bar{\theta }}^{c},A\left(\bar{\theta },P_{\theta_{0}}\right),\tau \left(\bar{\theta },P_{\theta_{0}}\right)\right)\;dP_{\theta_{0}}\left(x\right)+o_{P}\left(1\right)\\ \leq \int n^{c}\left(x,\bar{\theta },{\bar{t}}_{\bar{\theta }}^{c},A_{n}\left(\bar{\theta }\right),\tau_{n}\left(\bar{\theta }\right)\right)\;dP_{n}\left(x\right)-\int n^{c}\left(x,\bar{\theta },{\bar{t}}_{\bar{\theta }}^{c},A_{n}\left(\bar{\theta }\right),\tau_{n}\left(\bar{\theta }\right)\right)\;dP_{\theta_{0}}\left(x\right)+o_{P}\left(1\right)\\ \leq \underset{C}{\sup}\left|\int n^{c}\left(x,\theta ,t,A,\tau \right)\;dP_{n}\left(x\right)-\int n^{c}\left(x,\theta ,t,A,\tau \right)\;dP_{\theta_{0}}\left(x\right)\right|+o_{P}\left(1\right). \end{array}$$

Using (A45), (A49), and (A50), we deduce that $\sup_{\theta \in \Theta }∥{\bar{t}}_{\theta }^{c}-t_{\theta }^{c}\left(P_{\theta_{0}}\right)∥\to 0$ in probability. In particular, for large *n*, ${\bar{t}}_{\theta }^{c}\in \mathrm{int}\left(N_{t_{\theta }^{c}\left(P_{\theta_{0}}\right)}\right)$ uniformly in θ. Since $t\mapsto \int n^{c}\left(x,\theta ,t,A_{n}\left(\theta \right),\tau_{n}\left(\theta \right)\right)\;dP_{n}\left(x\right)$ is concave, the maximizer ${\hat{t}}_{\theta }^{c}$ belongs to $\mathrm{int}(N_{t_{\theta }^{c}\left(P_{\theta_{0}}\right)})$ for *n* sufficiently large. Then $\sup_{\theta \in \Theta }∥{\hat{t}}_{\theta }^{c}-t_{\theta }^{c}\left(P_{\theta_{0}}\right)∥\to 0$ in probability.

(2) For large *n*, we can write

(A51) $$\begin{array}{ccc} & & \underset{\theta \in \Theta }{\sup}\left|\int n^{c}\left(x,\theta ,{\hat{t}}_{\theta }^{c},A_{n}\left(\theta \right),\tau_{n}\left(\theta \right)\right)\;dP_{n}\left(x\right)-\int n^{c}\left(x,\theta ,t_{\theta }^{c}\left(P_{\theta_{0}}\right),A\left(\theta ,P_{\theta_{0}}\right),\tau \left(\theta ,P_{\theta_{0}}\right)\right)\;dP_{\theta_{0}}\left(x\right)\right|\\ & & =\underset{\theta \in \Theta }{\sup}\left|\int n^{c}\left(x,\theta ,{\bar{t}}_{\theta }^{c},A_{n}\left(\theta \right),\tau_{n}\left(\theta \right)\right)\;dP_{n}\left(x\right)-\int n^{c}\left(x,\theta ,t_{\theta }^{c}\left(P_{\theta_{0}}\right),A\left(\theta ,P_{\theta_{0}}\right),\tau \left(\theta ,P_{\theta_{0}}\right)\right)\;dP_{\theta_{0}}\left(x\right)\right|\\ & & :=\underset{\theta \in \Theta }{\sup}\left|B\right|. \end{array}$$

Note that

(A52) $$\begin{array}{cc} \int n^{c}(x, & \theta ,t_{\theta }^{c}\left(P_{\theta_{0}}\right),A_{n}\left(\theta \right),\tau_{n}\left(\theta \right))\;dP_{n}\left(x\right)-\int n^{c}\left(x,\theta ,t_{\theta }^{c}\left(P_{\theta_{0}}\right),A\left(\theta ,P_{\theta_{0}}\right),\tau \left(\theta ,P_{\theta_{0}}\right)\right)\;dP_{\theta_{0}}\left(x\right)\leq \\ & B\leq \int n^{c}\left(x,\theta ,{\bar{t}}_{\theta }^{c},A_{n}\left(\theta \right),\tau_{n}\left(\theta \right)\right)\;dP_{n}\left(x\right)-\int n^{c}\left(x,\theta ,{\bar{t}}_{\theta }^{c},A\left(\theta ,P_{\theta_{0}}\right),\tau \left(\theta ,P_{\theta_{0}}\right)\right)\;dP_{\theta_{0}}\left(x\right). \end{array}$$

In order to prove that $\sup_{\theta \in \Theta }\left|B\right|\to 0$ in probability, we first prove that

(A53) $$\underset{\theta \in \Theta }{\sup}\underset{t\in N_{t_{\theta }^{c}\left(P_{\theta_{0}}\right)}}{\sup}\left|\int n^{c}\left(x,\theta ,t,A_{n}\left(\theta \right),\tau_{n}\left(\theta \right)\right)\;dP_{n}\left(x\right)-\int n^{c}\left(x,\theta ,t,A\left(\theta ,P_{\theta_{0}}\right),\tau \left(\theta ,P_{\theta_{0}}\right)\right)\;dP_{\theta_{0}}\left(x\right)\right|\to 0\;$$

in probability. Note that

$$\begin{array}{ccc} & & \underset{\theta \in \Theta }{\sup}\underset{t\in N_{t_{\theta }^{c}\left(P_{\theta_{0}}\right)}}{\sup}\left|\int n^{c}\left(x,\theta ,t,A_{n}\left(\theta \right),\tau_{n}\left(\theta \right)\right)\;dP_{n}\left(x\right)-\int n^{c}\left(x,\theta ,t,A\left(\theta ,P_{\theta_{0}}\right),\tau \left(\theta ,P_{\theta_{0}}\right)\right)\;dP_{\theta_{0}}\left(x\right)\right|\\ & & \leq \underset{\theta \in \Theta }{\sup}\underset{t\in N_{t_{\theta }^{c}\left(P_{\theta_{0}}\right)}}{\sup}\left|\int n^{c}\left(x,\theta ,t,A_{n}\left(\theta \right),\tau_{n}\left(\theta \right)\right)\;dP_{n}\left(x\right)-\int n^{c}\left(x,\theta ,t,A_{n}\left(\theta \right),\tau_{n}\left(\theta \right)\right)\;dP_{\theta_{0}}\left(x\right)\right|\\ & & +\underset{\theta \in \Theta }{\sup}\underset{t\in N_{t_{\theta }^{c}\left(P_{\theta_{0}}\right)}}{\sup}\left|\int n^{c}\left(x,\theta ,t,A_{n}\left(\theta \right),\tau_{n}\left(\theta \right)\right)\;dP_{\theta_{0}}\left(x\right)-\int n^{c}\left(x,\theta ,t,A\left(\theta ,P_{\theta_{0}}\right),\tau \left(\theta ,P_{\theta_{0}}\right)\right)\;dP_{\theta_{0}}\left(x\right)\right|. \end{array}$$

The first term on the right-hand side of the above inequality tends to 0 in probability, using (A45) and Assumption 3. Regarding the second term, we have the convergence (A40), and combining this with Assumption 5.d, we obtain

(A54) $$\underset{\theta \in \Theta }{\sup}\underset{t\in N_{t_{\theta }^{c}\left(P_{\theta_{0}}\right)}}{\sup}\left|\int n^{c}\left(x,\theta ,t,A_{n}\left(\theta \right),\tau_{n}\left(\theta \right)\right)\;dP_{\theta_{0}}\left(x\right)-\int n^{c}\left(x,\theta ,t,A\left(\theta ,P_{\theta_{0}}\right),\tau \left(\theta ,P_{\theta_{0}}\right)\right)\;dP_{\theta_{0}}\left(x\right)\right|\to 0\;$$

in probability. Consequently, (A53) holds. Then, (A51) and (A52) lead to

(A55) $$\underset{\theta \in \Theta }{\sup}\left|\int n^{c}\left(x,\theta ,{\hat{t}}_{\theta }^{c},A_{n}\left(\theta \right),\tau_{n}\left(\theta \right)\right)\;dP_{n}\left(x\right)-\int n^{c}\left(x,\theta ,t_{\theta }^{c}\left(P_{\theta_{0}}\right),A\left(\theta ,P_{\theta_{0}}\right),\tau \left(\theta ,P_{\theta_{0}}\right)\right)\;dP_{\theta_{0}}\left(x\right)\right|\to 0.$$

Assumptions 5 (e) and (f) ensure that $\theta_{0}$ is well separated in the sense that ∀ε>0,

(A56) $$\begin{array}{ccc} & & \underset{\left\{\theta ;\;∥\theta -\theta_{0}∥\geq \varepsilon \right\}}{\inf}\int n^{c}\left(x,\theta ,t_{\theta }^{c}\left(P_{\theta_{0}}\right),A\left(\theta ,P_{\theta_{0}}\right),\tau \left(\theta ,P_{\theta_{0}}\right)\right)\;dP_{\theta_{0}}\left(x\right)\\ & & >0=\int n^{c}\left(x,\theta_{0},t_{\theta }^{c}\left(P_{\theta_{0}}\right),A\left(\theta_{0},P_{\theta_{0}}\right),\tau \left(\theta_{0},P_{\theta_{0}}\right)\right)\;dP_{\theta_{0}}\left(x\right). \end{array}$$

Finally, (A55) and (A56) imply that ${\hat{\theta }}_{\varphi }^{c}\to \theta_{0}$ in probability, on the basis of Theorem 5.7, p. 45 from [18]. □
